# Using mass cytometry for the analysis of samples of the human airways

**DOI:** 10.3389/fimmu.2022.1004583

**Published:** 2022-12-12

**Authors:** Marianne Rocha-Hasler, Lena Müller, Anja Wagner, Aldine Tu, Victoria Stanek, Nicholas James Campion, Tina Bartosik, Mohammed Zghaebi, Slagjana Stoshikj, Daniela Gompelmann, Andreas Zech, Henrik Mei, Klaus Kratochwill, Andreas Spittler, Marco Idzko, Sven Schneider, Julia Eckl-Dorna

**Affiliations:** ^1^ Allergology and Sinusitis Research Lab, Department of Otorhinolaryngology, Medical University of Vienna, Vienna, Austria; ^2^ Core Facility Flow Cytometry & Department of Surgery, Research Lab, Medical University of Vienna, Vienna, Austria; ^3^ Core Facility Proteomics, Medical University of Vienna, Vienna, Austria; ^4^ Division of Pediatric Nephrology and Gastroenterology, Department of Pediatrics and Adolescent Medicine, Comprehensive Center for Pediatrics, Medical University of Vienna, Vienna, Austria; ^5^ Division of Pulmonology, Department of Internal Medicine II, Medical University Vienna, Vienna, Austria; ^6^ German Rheumatism Research Center Berlin, Berlin, Germany

**Keywords:** mass cytometry, CyTOF, antibody titration, BALF, nasal polyp, tissue digestion, asthma, CRSwNP

## Abstract

Mass cytometry (MC) is a powerful method for mapping complex cellular systems at single-cell levels, based on the detection of cellular proteins. Numerous studies have been performed using human blood, but there is a lack of protocols describing the processing and labeling of bronchoalveolar lavage fluid (BALF) and nasal polyps (NP) for acquisition by MC. These specimens are essential in the investigation of immune cell characteristics in airway diseases such as asthma and chronic rhinosinusitis with NP (CRSwNP). Here we optimized a workflow for processing, labeling, and acquisition of BALF and NP cells by MC. Among three methods tested for NP digestion, combined enzymatic/mechanical processing yielded maximum cell recovery, viability and labeling patterns compared to the other methods. Treatment with DNAse improved sample acquisition by MC. In a final step, we performed a comparison of blood, BALF and NP cell composition using a 31-marker MC antibody panel, revealing expected differences between the different tissue but also heterogeneity among the BALF and NP samples. We here introduce an optimized workflow for the MC analysis of human NP and BALF, which enables comparative analysis of different samples in larger cohorts. A deeper understanding of immune cell characteristics in these samples may guide future researchers and clinicians to a better disease management.

## 1 Introduction

Mass cytometry (MC), or cytometry by time of flight (CyTOF), is a high-dimensional single-cell approach using antibodies labelled with non-radioactive heavy metal isotopes to detect intracellular or surface molecules (1, 2). This technique allows for the analysis of up to 60 different parameters in parallel, thus enabling the investigation of numerous specific cell populations and subpopulations in a single measurement (2). Furthermore, algorithms for data analysis through dimensionality reduction and clustering are an unbiased approach providing deep insights into the relationship among the different cell types present in a single sample (3). Therefore, this method could promote the discovery of novel biomarkers guiding prognosis and therapies of different diseases (4, 5).

In comparison to the widely used flow cytometry, MC presents many advantages. These include not only the larger number of parameters that can be analyzed with higher resolution, but also the absence of auto-fluorescence, low to no need for compensation, and the minimal spillover that can be achieved due to the purity of metal tags and the careful panel design (1, 6). However, MC also has some disadvantages, such as a higher cell loss during the experimental procedure, longer acquisition time, and the inability of recovering live cells at the end, due to their atomization and ionization during acquisition (7, 8).

MC has broad applications, including immune phenotyping, analysis of transcription factors, cell cycle and proliferation analysis (9, 10). Immune cell phenotyping using more than 30 markers has been performed in human blood samples for more than a decade. However, this technique has not yet been widely adapted to tissue derived samples such as the ones originated from the human airways (11, 12). Nevertheless, investigating and comparing immune cell composition in airway derived cells of both healthy subjects and patients suffering from different airway diseases (e.g., chronic sinusitis with nasal polyps [CRSwNP] or asthma) is central to better understand the underlying pathophysiological mechanisms. Tissue derived samples can provide more detailed and complementary information as compared to blood samples alone (12, 13).

Regarding airway conditions, CRS is a complex inflammatory airway disease that encompasses a broad spectrum of clinical variants, including CRSwNP, which corresponds to the diverse immunocellular profile underlying it (14). Asthma is a chronic inflammatory disease of the airways with reversible episodic obstruction which presents an extensive heterogeneity of cell types (13). Both conditions, especially when they are comorbid in a patient, represent an intricate immunocellular landscape and thus patient’s therapy management is often challenging. Mostly the investigations rely on flow cytometry analysis of blood or peripheral blood mononuclear cells (PBMCs), but MC has demonstrated its potential to increase the level of details of the cell markers in a population (15).

In this respect, MC has been applied to analyze cells isolated from bronchoalveolar lavage fluid (BALF) of COPD or sarcoidosis patients (16, 17). To the best of our knowledge, to date, only one study has used MC for analysis of CRSwNP. This study revealed several unique innate and T cell populations in NP as compared to healthy mucosa, whilst results from flow cytometry confirmed that both techniques detected similar proportions of major cell subsets (12). Thus, MC is a powerful tool which could also enhance our understanding of the complex relationships between CRS and asthma. However, standardized protocols allowing for processing and high-quality acquisition for airway derived samples are missing so far and are crucial for reproducibility and comparability of results (8). Thus, the present study aimed to establish protocols for optimal processing, labeling, and acquisition of cells from human NP and BALF for MC. After applying the optimized protocols, human NP, BALF and blood samples were stained using the commercially available Maxpar Direct Immune Profiling Assay (MDIPA) as a backbone, including three additional markers in a final validation step.

## 2 Material and methods

### 2.1 Nasal polyp digestion

Three different digestion protocols were tested for processing the NPs. The specimens were weighed, split into three similar parts (same weight of maximum 0.5 g) and each of them digested using one of the three different procedures described below.

#### 2.1.1 Collagenase

The NP piece was minced in a sterile polypropylene petri dish and transferred to a 50 mL conical tube containing 2 mg/mL of Collagenase IV (Gibco, Thermo Fisher Scientific), 25 µg/mL DNAse (Sigma-Aldrich), in RPMI1640 (Gibco, Thermo Fisher Scientific) (18). After incubation for 40 min at 37°C in a water bath, the sample was passed through a 30 µm cell strainer (Miltenyi Biotec, Bergisch Gladbach, Germany) into a new 50 mL conical tube, using a 20 mL syringe stopper. The cell strainer was then washed with 1 mL of RPMI1640, and the cell suspension centrifuged at 528 x *g* for 5 min at room temperature (RT). The supernatant was removed before proceeding to cell labeling for MC.

#### 2.1.2 Protease

The NP fragment was minced on a sterile polypropylene petri dish and put into a 50 mL conical tube containing 1 mL of dissociation buffer (DPBS [Gibco, Thermo Fisher Scientific], containing 10 mg/mL protease from Bacillus Licheniformis [Sigma-Aldrich], and 0.5 mM EDTA [Sigma-Aldrich]) as described (19). After one hour of incubation on ice, 200 µL inactivation buffer (2% BSA [Sigma-Aldrich] prepared in HBSS) were added and the cell suspension was centrifuged at 4°C with 400 x *g* for 5 min. The cells were resuspended in 200 µL of wash buffer (1% BSA prepared in HBSS) and passed through a 30 µm cell strainer into a new 50 mL conical tube, using a 20 mL syringe stopper. The cell strainer was then washed with 1 mL of HBSS, and the cell suspension centrifuged at 400 x *g* for 5 min at 4°C. The supernatant was removed before proceeding to cell labeling for MC.

#### 2.1.3 Combined enzymatic and mechanical dissociation

The NP fragment was minced on a sterile polypropylene petri dish and placed in a GentleMACS C tube (Miltenyi Biotec) containing 2.35 mL serum-free MEM (Sigma-Aldrich) containing 1% penicillin/streptomycin solution (Gibco, Thermo Fisher Scientific), 100 µL of enzyme D, 50 µL of enzyme R, and 12.5 µL of enzyme A (Multi Tissue Dissociation Kit 1, Miltenyi Biotec) according to the manufacturer’s protocol. The C tube was incubated running the protocol 37C_Multi_B (duration 61 min) on the GentleMACS Octo Dissociator with Heaters (Miltenyi Biotec). After the protocol was completed, the cell suspension was mixed by pipetting and passed through a 30 µm cell strainer into a new 50 mL conical tube, with the help of a 20 mL syringe stopper. The cell strainer was washed with 15 mL MEM supplemented with 1% penicillin/streptomycin medium and 10% FBS was added to the cell suspension. The total volume was mixed by pipetting up and down and centrifuged at 300 x *g* for 7 min at RT. The supernatant was removed before proceeding to cell labeling for MC.

### 2.2 Mass cytometry assay

Antibody labeling for MC was performed as described before (20) with the following modifications.

#### 2.2.1 Whole blood

400 µL fresh heparinized whole blood was resuspended in 1 mL of Maxpar Cell Staining Buffer (CSB) (Standard BioTools, South San Francisco, California, USA) supplemented with 10 µL of 10 KU/mL heparin solution (Sigma-Aldrich, St. Louis, Missouri, USA) and incubated at RT for 20 min. Meanwhile, live cells were counted after Trypan Blue staining (Sigma-Aldrich) using a Neubauer chamber, and the total amount of 3 x 10^6^ cells/mL was used for staining. After incubation with heparin, 2 mL of CSB were added to the tube and gently mixed. The cell suspension was centrifuged at 300 x *g* for 7 min at RT. Thereafter the supernatant was removed. The pellet was resuspended in 270 µL of CSB and 30 µL of an antibody master mix containing CD3-^154^Sm, CD11b/Mac-1-^209^Bi, CD45-^112^Cd, DNA intercalator Cell-ID ^103^Rh (all from Standard BioTools) (**Supplementary Table 1**), diluted in CSB, was added. The cell suspension was mixed and incubated for 30 min at RT. 250 µL of Cal-Lyse lysing solution (Life Technologies, Carlsbad, California, USA) was added, mixed, and incubated in the dark for 10 min at RT. 3 mL of de-ionized water (DIW) (B. Braun, Melsungen, Germany) was added to the tube and again incubated in the dark for 10 min at RT. The translucent cell suspension was centrifuged at 300 x *g* for 7 min at RT, the supernatant was removed, and the pellet was washed twice with 3 mL DIW. Then, the cell pellet was resuspended in 1 mL of fresh 1.6% formaldehyde solution (Thermo Fisher Scientific, Waltham, Massachusetts, USA), and incubated for 10 min at RT. Then, the cell suspension was centrifuged at 800 x *g* for 7 min at RT. The supernatant was discarded, the pellet was resuspended in DNA intercalator solution (1 mL of Fix-Perm buffer supplemented with 1 µL^191^Ir/^193^Ir DNA intercalator, all Standard BioTools), and incubated for 30 min at RT. After a last centrifugation step at 800 x *g* for 7 min at RT, 800 µL of the supernatant was discarded and the pellet was resuspended in the remaining 200 µL of supernatant. The final volume was divided into two aliquots of 100 µL which were stored at -80°C for no longer than two weeks. The samples were acquired on a Helios^®^ mass cytometer, Standard BioTools). Setup and tuning were performed according to the manufacturer’s instructions; cells were acquired with a speed of 250-350 events/second.

#### 2.2.2 Nasal polyp and BALF

After NP digestion described below or after receiving fresh BALF, cell suspensions were centrifuged at 300 x *g* for 5 min at RT. The supernatant was removed, and the pellet was resuspended in 1 mL CSB with 10 µL of 10 KU/mL heparin solution, then incubated for 20 min at RT. During this time, cells were counted after Trypan Blue staining and the total of 3 x 10^6^ cells was used for staining, as recommended in the standard protocol. After incubation with heparin, the cell suspension was centrifuged at 300 x *g* for 7 min at RT, the supernatant removed, the pellet resuspended in 50 µL of CSB with 5 µL of Human TruStain FcX (Biolegend, PerkinElmer, San Diego, California, USA), and incubated for 10 min at RT. Then, 215 µL of CSB was added to the tube, along with 30 µL of an antibody master mix containing CD3-^154^Sm, CD11b/Mac-1-^209^Bi, CD45-^112^Cd, DNA intercalator Cell-ID ^103^Rh, diluted in CSB. The suspension was mixed and incubated for 30 min at RT. After incubation, 3 mL of CSB was added and the cell suspension was centrifuged at 300 x *g* for 7 min at RT. The supernatant was removed and this last CSB wash step was repeated. After that, the cell pellet was resuspended in 1 mL fresh 1.6% formaldehyde solution, and incubated for 10 min at RT. The supernatant was discarded, the cell suspension was centrifuged at 800 x g for 7 min at RT, the pellet was resuspended in the intercalation solution (1 mL of Fix-Perm with 1 µL of DNA intercalator/Cell-ID ^191^Ir/^193^Ir), and incubated for 30 min at RT. After a last centrifugation step at 800 x *g* for 7 min at RT, 800 µL of the supernatant was discarded and the pellet was resuspended in the 200 µL left of supernatant. The final amount was divided into two aliquots of 100 µL which were stored at -80°C for no longer than two weeks. The samples were acquired on a Helios^®^ instrument as described above.

### 2.3 Removal of debris before sample labeling

Debris and dead cell removal was performed on blood, BALF and NP samples to improve the quality of the cell suspensions and counteract clogging of the Helios^®^ instrument, using either DNAse (Nuclease Universal Pierce™ solution 250 U/µL, Thermo Fisher Scientific), Basic MicroBeads (Miltenyi Biotec), or both combined.

#### 2.3.1 DNAse treatment

400 µL of blood or 3 x 10^6^ BALF or NP cells were resuspended in 1 mL of 0.01% DNAse solution in CSB. The sample was then incubated at 37°C for 30 min with 300 rpm on a Thermomixer (Eppendorf, Hamburg, Germany). Finally, the sample was mixed and transferred to a sterile 5 mL round-bottom polypropylene test tube, filled up with 1 mL CSB and centrifuged at 300 x *g* for 7 min at RT. The supernatant was removed before proceeding to cell labeling for MC.

#### 2.3.2 Basic MicroBeads

400 µL of blood or 3 x 10^6^ of BALF or NP cells were centrifuged at 300 x *g* for 10 min at RT. The Basic MicroBeads (Miltenyi Biotec) protocol was performed according to the manufacturer’s instructions. Briefly, the supernatant was removed, and the cell pellet resuspended in 50 µL of PBS (pH 7.2, Gibco, Thermo Fisher Scientific, supplemented with 0.5% BSA and 2 mM EDTA). 5 µL of pre-diluted Basic MicroBeads (1:10 with PBS) were added to the total cells, mixed, and incubated at 4°C for 25 min. 1 mL of buffer was added, the suspension was mixed and centrifuged at 300 x *g* for 10 min at RT. The supernatant was removed using a pipet, the pellet was resuspended in 500 µL buffer, and the cell suspension was applied onto pre-rinsed (with 2 mL buffer) LD columns (Miltenyi Biotec) placed on a QuadroMACS™ Separator (Miltenyi Biotec) and collected into a new 50 mL conical tube. The column was washed twice with 1 mL buffer and the total effluent collected in the same tube was spun down at 300 x *g* for 7 min at RT. The supernatant was removed before proceeding to cell labeling for MC.

#### 2.3.3 Basic MicroBeads and DNAse treatment combined

The same protocol described above for Basic MicroBeads was performed and after the centrifugation of the effluent at 300 x *g* for 7 min at RT, the supernatant was removed, the pellet was resuspended in 1 mL of the DNAse solution prepared and the respective protocol was performed as described above.

### 2.4 Antibody conjugation and titration

The Maxpar Direct Immunoprofiling Assay (MDIPA) antibody panel (Standard BioTools) was extended with three additional antibodies: anti-human CD11b/Mac-1-^209^Bi (Standard BioTools), anti-human CD23 and anti-human FcϵRI (both Biolegend, PerkinElmer, San Diego, California, USA). Anti-CD23 was labelled in-house with ^159^Tb using the Maxpar X8 kit and anti-FcϵRI with ^116^Cd using the Maxpar MCP9 kit, according to the manufacturer’s protocols. Labeling reaction with serial dilutions of antibodies were performed on blood, BALF and NP cells separately. After labeling protocol was performed, the cell suspensions were frozen in -80°C for maximum two weeks and thawed according to the manufacturer’s instructions (briefly described in 2.6).

All the antibodies were titrated for blood, BALF and NP, the stain index was calculated, the percentage of antibody positive cells and their adequate concentration to be used for labeling was determined.

After titration, the stain index was calculated using the formula Stain Index = ((Median of Positive – Median of Negative)/Standard Deviation of Negative * 2) (21) using FCS Express™ 7 (*De Novo* Software, Pasadena, California, USA) and FlowJo v.10.7.2 (BD Biosciences, Franklin Lakes, New Jersey, USA). The concentration of the antibody to be used in the full panel was determined.

### 2.5 Labeling with (extended) MDIPA

The full panel was validated by three independent experiments using fresh blood, NP and BALF. The processing and labeling followed the described methods in 2.2.1 for blood and 2.2.2 for NP and BALF cells, with a difference on the antibody labeling, as detailed below (for a detailed step-by-step protocol, please refer to supplementary methods):

Regarding the blood, after the heparin incubation and subsequent wash with CSB, the cell pellet was resuspended in 270 µL of CSB, the suspension was added to the lyophilized pellet of antibody mix pellet provided in the MDIPA kit and carefully mixed with the pipet. 30 µL of an antibody master mix containing the newly titrated antibodies anti-CD11b, anti-CD23, and anti-FcϵRI in CSB was immediately added to the cell suspension and again carefully mixed with the pipet. This suspension was incubated for 30 min at RT and the protocol followed as in 2.3.1.

As to the NP and the BALF cells, after the incubation with Human TruStain FcX and subsequent wash with CSB, the cell pellet was resuspended in 270 µL of CSB, the suspension was added to the lyophilized pellet of antibody mix provided in the MDIPA kit and carefully mixed with the pipet. 30 µL of an antibody master mix containing the newly titrated antibodies anti-CD11b, anti-CD23, and anti-FcϵRI in CSB was immediately added to the cell suspension and again carefully mixed with the pipet. This suspension was incubated for 30 min at RT and the protocol followed as in 2.3.2.

### 2.6 Mass cytometry

Immediately before sample acquisition on the Helios^®^ instrument, cells were washed twice with CSB and twice with Maxpar Cell Acquisition Solution (CAS) (Standard BioTools), by centrifugation at 800 x *g* for 7 min at RT. During the last step, the nucleated cells were counted after staining with 1:20 Trypan Blue using a Neubauer Chamber. The cells were resuspended in CAS supplemented with a 1:10 dilution of the EQ Four Element calibration beads (Standard BioTools), for 5 x 10^5^ – 1 x 10^6^ cells/mL. The suspension of beads and cells was filtered twice into a new 30 μm cell strainer cap tube (BD). The cell acquisition rate was kept between 150-350 cells per second. For testing of NP digestion protocols and DNAse and Basic MicroBead protocols 100,000 events were recorded, while for the validation of the labeling with the full antibody panel 400,000 events were acquired from each sample.

The files obtained were stored in *.fcs format. Individual FCS files from interrupted measurements were concatenated using the CyTOF Software v.7.0 (Standard BioTools).

### 2.7 Data analysis

After acquisition (but before concatenation), data were normalized using the CyTOF Software v.7.0 as described (22). Data of undesired events (dead cells, debris, normalization beads, true aggregates, and coincident ion clouds) were removed from raw data by manually gating out beads, and according to residual, center, offset, width, event length, DNA intercalator signals in biaxial plots vs Time parameter by gaussian discrimination (**Supplementary Figure 1**). This data clean-up was described before (22), and presently FCS Express™ 7 was used. The percentages of the gated populations in each channel were exported to GraphPad Prism Version 9 (GraphPad Software, San Diego, USA) to create graphs.

Analysis of antibody titration data was performed in FlowJo v.10.7.2 (BD). Dimensionality reduction was performed using opt-SNE in Cytobank (Beckman Coulter, Brea, California, USA). The analysis was done on all the three types of samples separately (specimens were not mixed for comparative analysis), after data cleanup as described above, on gated leukocytes CD45+CD66b- (excluding granulocytes). Thus, 31 channels were selected for running the algorithm: CD3, CD4, CD8a, CD11b, CD14, CD16, CD19, CD20, CD23, CD25/IL-R2a, CD27, CD28, CD38, CD45RA, CD45RB, CD45RO, CD56/NCAM, CD57, CD123/IL-3R, CD127/IL-7Ra, CD161, CD183/CXCR3, CD185/CXCR5, CD194/CCR4, CD196/CCR6, CD197/CCR7, CD294, FcϵRI, HLA-DR, IgD, TCRgd. For blood and BALF, the advanced settings were the default of the software, and the scales were normalized. For NP, the advanced settings were the default of the software, except the maximum iterations were modified to 500, and the scales were also normalized.

## 3 Results

### 3.1 Nasal polyp enzymatic/mechanical dissociation yields high number of viable cells for acquisition by mass cytometry

**Figure 1:**
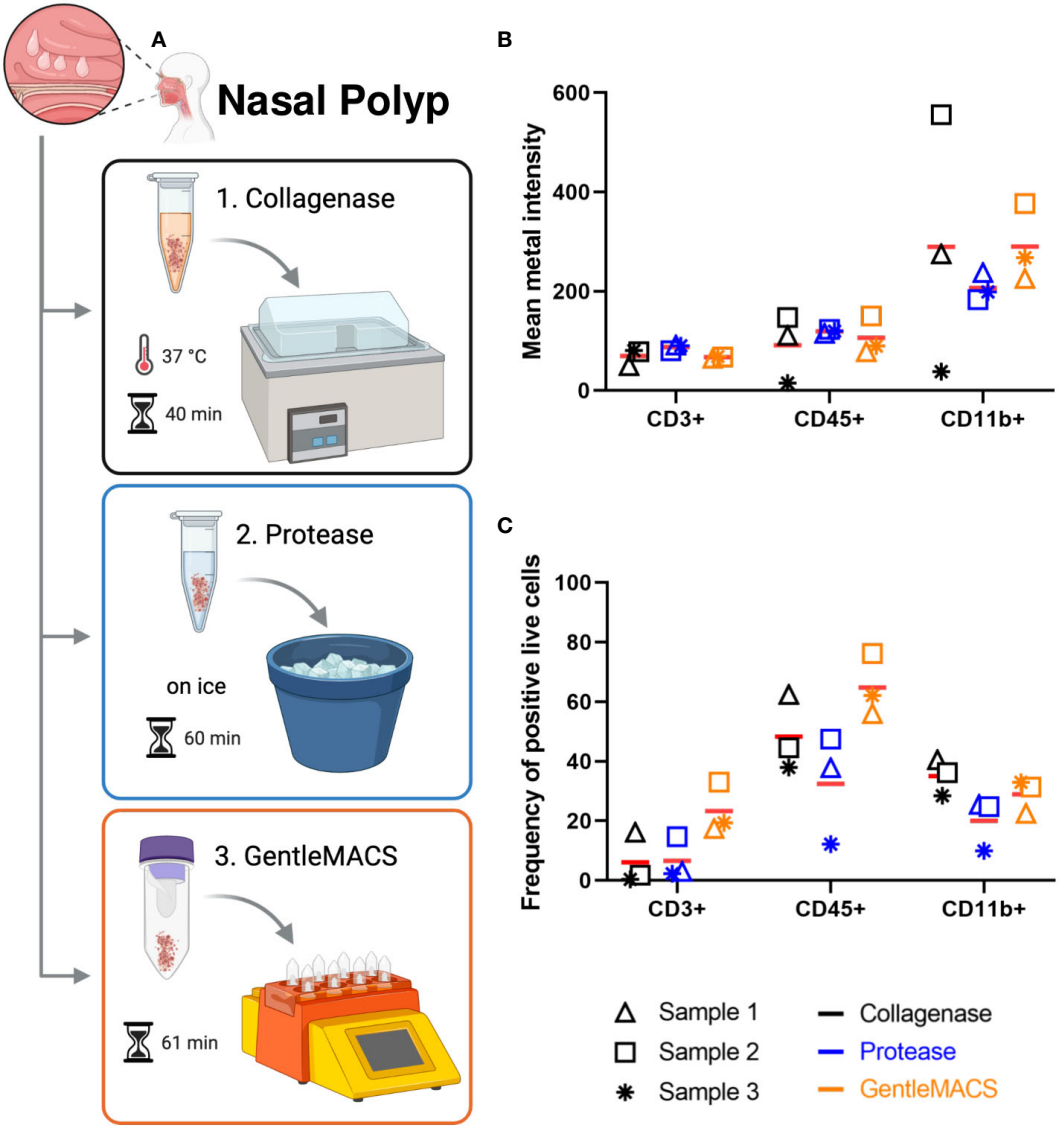
Nasal polyp digestion protocols for mass cytometry. **(A)** Nasal polyps were minced, and submitted to one of the following digestion protocols: 1. incubation at 37°C in a water bath for 40 min in collagenase solution; 2. incubation on ice for 60 min in protease solution; and 3. incubation for 53 min in enzyme mix (A, D, and R enzymes) in GentleMACS^®^ (Miltenyi). Single cell suspensions obtained were used for labeling according to the Maxpar Direct Immune Profiling Assay (MDIPA) protocol (figure created with BioRender.com). **(B)** Mean metal intensity (MMI) of CD3, CD45 and CD11b positive live cells obtained with each protocol by mass cytometry. Bars represent mean of three independent experiments. **(C)** Percentage of CD3, CD45 and CD11b positive live cells obtained with each protocol by mass cytometry. Lines represent mean of three independent experiments.

First, we tested protocols for preparation of single cell suspensions from NP that would result in the highest number of viable cells for subsequent labeling. Thus we tested three commonly used protocols for tissue digestion using collagenase (18), protease (19) or combined enzymatic/mechanical digestion (**Figure 1A**). Cell counts determined after digestion and before labeling revealed that tissue digestion with GentleMACS or collagenase provided comparable cell yield, while cell suspensions prepared with protease yielded lower cell number (**Supplementary Figure 2**). Furthermore, the cell viability determined by the intercalator Cell-ID ^103^Rh dead cell detection assay by MC obtained was lowest with collagenase as compared to the other methods. In general, the triplicates of the protocols resulted in variable cell count despite using comparable sample weight, especially for collagenase and protease methods (**Supplementary Figure 2A**), which did not seem to impact on cell viability, except for one of the experiments with collagenase (**Supplementary Figure 2A**). Analysis of CD3, CD45 and CD11b expression in live cells were performed in all three protocols (**Supplementary Table 1** and **Supplementary Figure 2**). Here, though the expression levels as determined by mean metal intensity (MMI) was comparable between the different protocols (**Figure 1B**), preparation of cell suspensions by combined enzymatic/mechanical digestion resulted in the highest percentage of CD3+ (23 ± 9%) and CD45+ (65 ± 10%) cells (**Figure 1C**; **Supplementary Figure 2**). Based on these results, all further experiments with NP were performed using combined enzymatic/mechanical digestion.

### 3.2 Higher cell count and viability of BALF cells upon preprocessing with DNAse than with Basic MicroBeads

**Figure 2:**
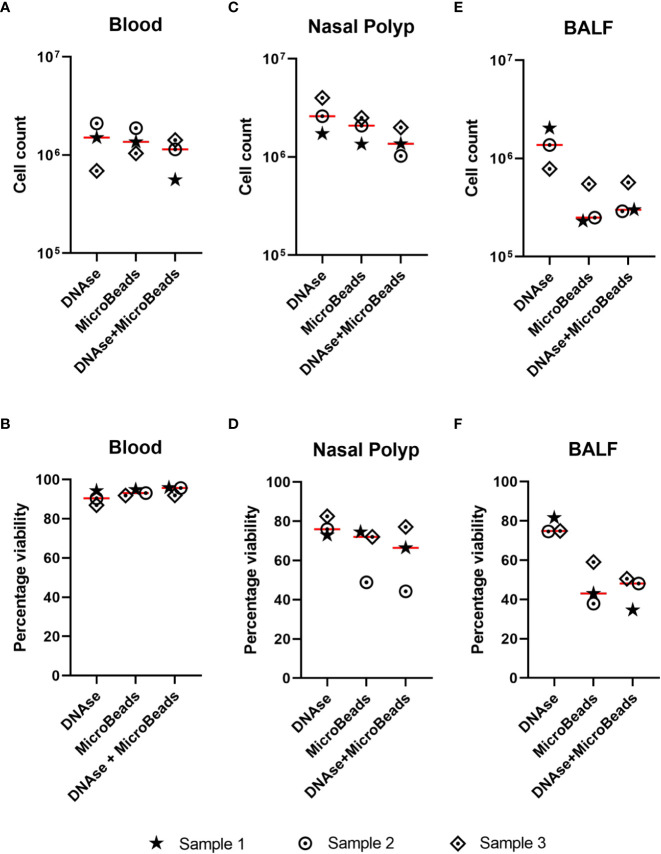
Effect of DNAse and/or microbeads on cell count and viability in different sample types. **(A-F)** Single cell suspensions were treated with either DNAse solution, Basic MicroBeads (Miltenyi), or combined DNAse solution + Basic MicroBeads (Miltenyi), prior to labeling according to Maxpar Direct Immune Profiling Assay protocol. The upper graphs show the cell count after treatment for (total number, y-axis) **(A)** blood **(B)** nasal polyp and **(C)** bronchoalveolar lavage fluid (BALF) and lower graphs show the percentage of cell viability upon acquisition (given in percentage, y-axis) for **(D)** blood, **(E)** nasal polyp, and **(F)** BALF using the three above mentioned treatments. Lines represent mean of three independent experiments.

As initial MC acquisitions of NP cell suspensions showed clogging more frequently than blood samples, we tested DNAse solution and Basic MicroBeads, separately and in combination, to minimize cell clumping mediated by DNA released from dead or dying cells, or to remove debris and dead cells, respectively. As described in detail in Methods, all treatments were applied to 3 x 10^6^ cells/mL. In blood samples, the mean cell counts were 1.4 x 10^6^ cells/mL after DNAse application, 1.4 x 10^6^ cells/mL after the use of Basic MicroBeads, and 1.0 x 10^6^ cells/mL after applying both treatments combined (**Figure 2A**). The corresponding mean cell viabilities were 91 ± 4%, 93 ± 2%, and 95 ± 2% respectively (**Figure 2B**). Similarly, the three different preprocessing protocols were showing a comparable trend in NPs regarding cell counts (DNAse: 2.78 x 10^6^ ± 1.15 x 10^6^, Basic MicroBeads: 1.98 x 10^6^ ± 0.58 x 10^6^, and DNAse+Basic Microbeads: 1.46 x 10^6^ ± 0.49 x 10^6^ cells/mL **Figure 2C**) and viability (77 ± 5%, 65 ± 14%, and 63 ± 17% respectively, **Figure 2D**). As for the BALF, the mean cell count was 1.39 x 10^6^ ± 0.62 x 10^6^ cells/mL after DNAse treatment, while Basic MicroBeads yielded 3.43 x 10^5^ ± 1.79 x 10^5^ cells/mL on average, and both protocols combined resulted in a mean cell count of 3.9 x 10^5^ ± 1.6 x 10^5^ cells/mL (**Figure 2E**). A similar tendency was observed in mean percentage of cell viability with respects to the different protocols used: 77 ± 4% for DNAse, 47 ± 11% for Basic MicroBeads, and 44 ± 9% for DNAse + Basic MicroBeads, respectively (**Figure 2F**). During acquisition, we experienced fewer clogging events (perceived by increase in argon gas pressure (in pounds per square inch (psi)), as well as by the variability on the speed of sample injection (in µL/sec) in samples where DNAse was used as compared to those prepared with Basic MicroBeads alone (data not shown). Based on this observation and the fact that DNAse treatment alone yielded higher cell count and viability for BALF, we decided to include DNAse treatment in our sample processing protocol. Though only minor differences between preprocessing protocols were observed in NPs and blood, we also included DNAse treatment for the processing of these tissues for consistency.

### 3.3 Titration of additional antibodies anti-human CD11b, CD23 and FcϵRI

**Figure 3:**
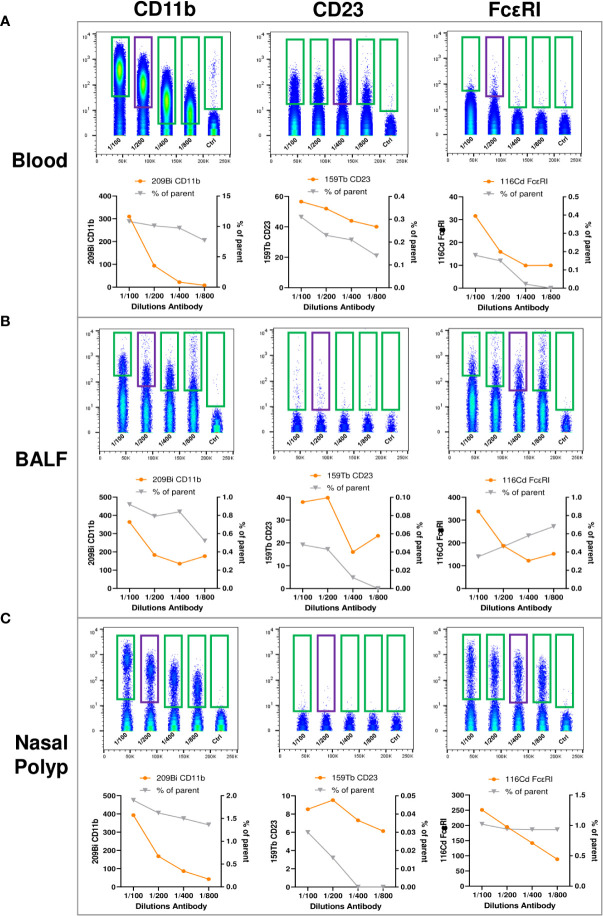
Titration of anti-human CD11b, anti-human CD23, and anti-human FcϵRI antibodies for expansion of the MDIPA panel. **(A-C)**, **(A)** Blood, **(B)** bronchoalveolar lavage fluid (BALF) and **(C)** nasal polyp were stained with serial dilutions (1:100, 1:200, 1:400, 1:800 and unstained control) of ^209^Bi anti-human CD11b (left panels), ^159^Tb anti-human CD23 (middle panel) and ^116^Cd anti-human FcϵRI (right panels). Lower panels show mean metal intensity (left y-axis) and percentage of cells positive for the respective antibody (right y-axis).

To gain a broad overview of immune cell subpopulations present in blood, NP and BALF, tailored to the analysis of type 2 immunopathology we used the commercially available MDIPA antibody panel as a backbone (20, 23), and integrated antibodies labeling the high and low affinity receptors for IgE (FcϵRI and CD23, respectively), and the macrophage marker CD11b (**Supplementary Table 2**). These three antibody conjugates were titrated using blood, BALF and NP cells to determine the optimal labeling concentration for all three tissues, by testing different dilutions for labeling (**Figures 3A–C**). Based on stain index and percentage of stained cells, we considered a dilution of 1:200 for CD11b as optimal for all three sample types. For the CD23 antibody the dilution of 1:400 was selected for blood, and 1:200 for BALF and for NP cells; for the FcϵRI antibody a dilution of 1:200 in blood and 1:400 in BALF and NP cells.

### 3.4 Staining with extended MDIPA panel and data analysis using dimensionality reduction algorithm

**Figure 4:**
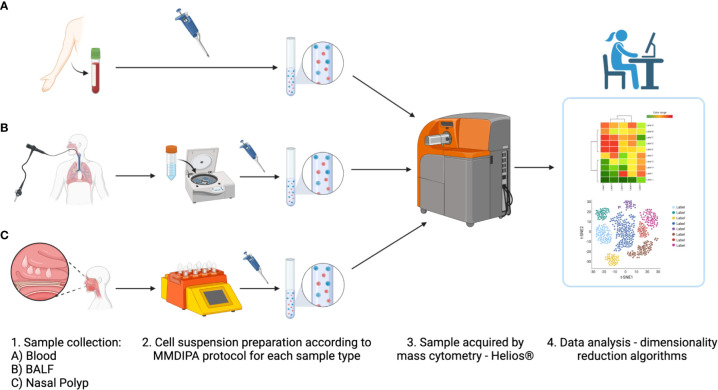
Overview of modified labeling protocol with Maxpar Direct Immune Profiling Assay (MDIPA) panel for mass cytometry (MMDIPA) using human **(A)** blood, **(B)** bronchoalveolar lavage fluid (BALF), and **(C)** nasal polyp. (1) After collection, (2) samples were worked up following the MMDIPA protocol established. (3) The obtained single cell suspensions were acquired in a Helios^®^ (Standard BioTools), and (4) the data were analysed using selected softwares (figure created with BioRender.com).

**Figure 5:**
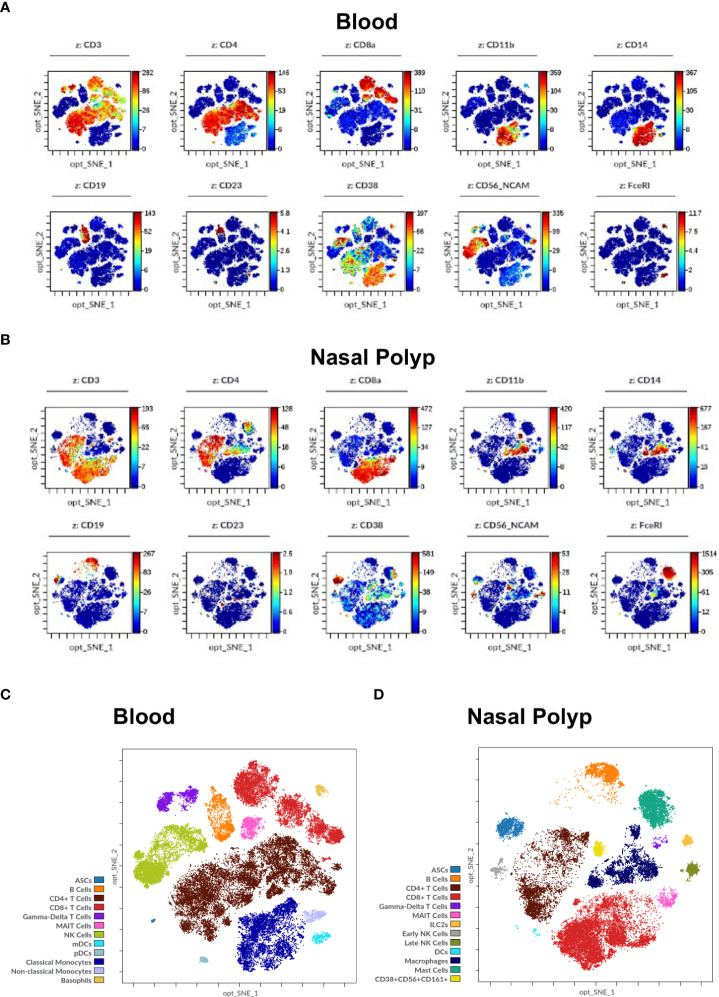
Immune cell markers in human blood and nasal polyp. **(A, B)** Opt-SNE plots show selected markers of the modified Maxpar Direct Immune Profiling Assay (MMDIPA panel), namely CD3, CD4, CD8a, CD11b, CD14, CD19, CD23, CD38, CD56, and FcϵRI in **(A)** Blood and **(B)** nasal polyp single cell suspensions acquired in Helios^®^ (Standard BioTools). The scales vary throughout the plots according to the intensity presented by the markers. Plots shown are representative of three independent experiments. **(C)** Identification of immune cell populations in blood using mass cytometry. Opt-SNE show manually selected cell populations: Antibody Secreting Cells (ASCs), B Cells, CD4+ T Cells, CD8+ T Cells, Gamma-Delta T Cells, Mucosal-associated invariant T (MAIT) Cells, Natural Killer (NK) Cells, Myeloid Dendritic Cells (mDCs), Plasmacytoid Dendritic Cells (pDCs), Classical Monocytes, Non-classical Monocytes, and Basophils. **(D)** Identification of immune cell populations in nasal polyp using mass cytometry. Opt-SNE s show manually selected cell populations: ASCs, B Cells, CD4+ T Cells, CD8+ T Cells, Gamma-Delta T Cells, MAIT Cells, Type 2 Innate Lymphoid Cells (ILC2s), NK Cells, Dendritic Cells (DCs), Macrophages, Mast Cells, CD38+CD56+CD161+ Cells. The manually annotated populations in **(C)** and **(D)** are overlaid as a color-dimension.

**Figure 6:**
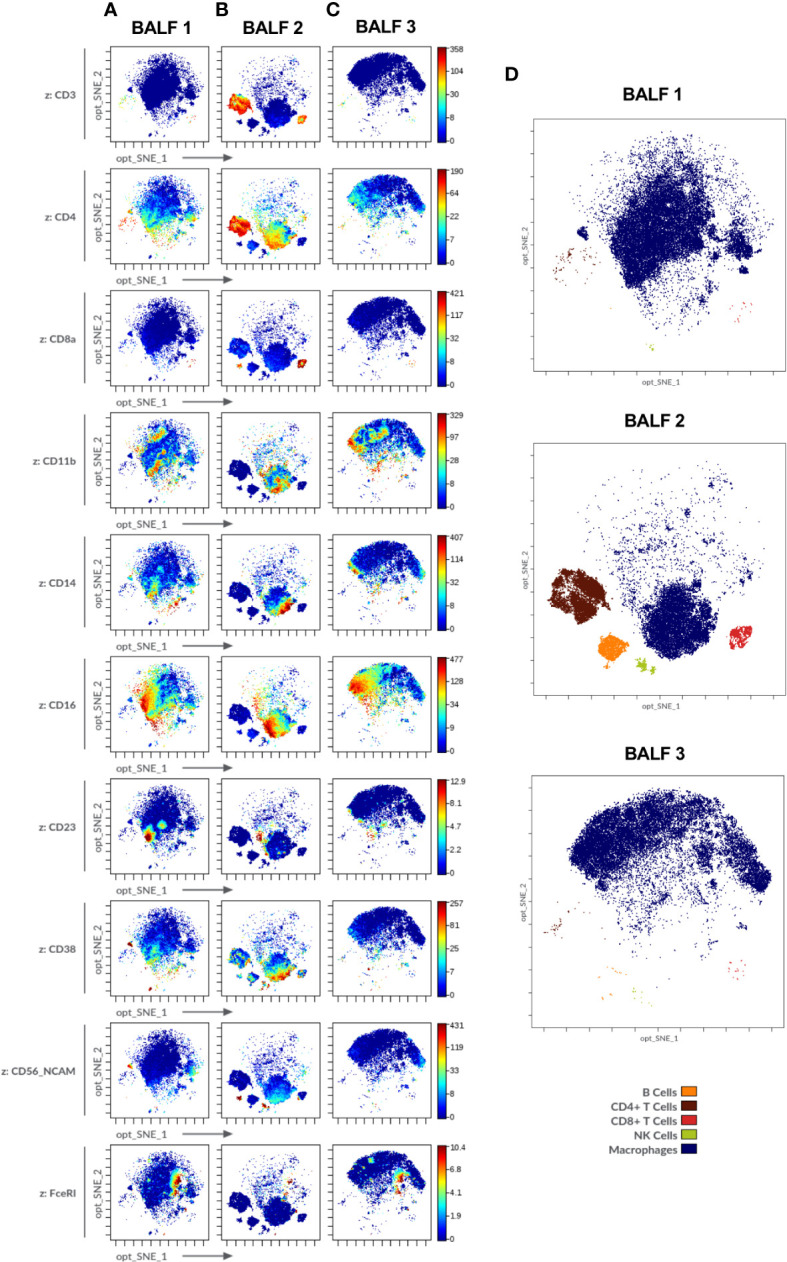
Immune cell markers in human bronchoalveolar lavage fluid (BALF) of diverse patients acquired in Helios^®^ (Standard BioTools). **(A-C)** Opt-SNE plots show selected markers of the modified Maxpar Direct Immune Profiling Assay (MMDIPA panel) namely CD3, CD4, CD8a, CD11b, CD14, CD16, CD23, CD38, CD56, and FcϵRI. BALFs from patients with **(A)** non-small cell lung carcinoma (BALF 1), **(B)** previous COVID-19 infection (BALF 2) and **(C)** asthma (BALF 3) are shown. Plots shown are representative of three independent experiments. **(D)** Identification of immune cell populations in the three BALF using mass cytometry. Opt-SNE show manually selected cell populations: B Cells, CD4+ T Cells, CD8+ T Cells, Natural Killer (NK) Cells, Macrophages. The manually annotated populations in **(C)** and **(D)** are overlaid as a color-dimension.

After optimizing sample processing, handling and antibody dilutions were optimized, we validated the workflow and labeling protocol, on blood, NP and BALF samples (**Figures 4**, **5**). After data cleanup (**Supplementary Figure 1**) sample data were analyzed in Cytobank using opt-SNE which is an unsupervised algorithm that provides a broad visualization of events in 2-dimensional map of multidimensional data and allows reproducibility of the analysis. Overall, all major leukocyte subsets were identified in all sample types according to their canonical phenotypes, such as T cells (CD3+), including CD4+ helper T cells (blood 40.3 ± 7.9%; NP 20.5 ± 9.6%) and CD8+ cytotoxic T cells (blood 16.1 ± 8.7%; NP 24.8 ± 10.3%), monocytes (CD14+) (blood 18.1 ± 2.7%), and B cells (CD19+) (blood 5.5 ± 2.4%; NP 8.9 ± 7.2%) (**Figures 5**, **6**, additional markers: **Supplementary Figures 3–5**; percentage of major immune cell subpopulations: **Supplementary Tables 3**). Additionally, we identified ILC2 cells (Lin^-^, CD127^+^, CD294^+^, CD161^+^) in NPs, and an NK cell population expressing CD161. Furthermore, the additional markers CD23 and FcϵRI enable the identification of cell expressing IgE receptors, while CD11b improved the accuracy of the determination of monocytes, NK cells, dendritic cells, and macrophages, according to the specimen in question (blood, BALF or NP).

For BALF analysis, samples derived from three patients suffering from different airway diseases were chosen: BALF 1-non-small cell lung carcinoma, BALF 2-post-COVID-19, BALF 3-asthma (**Figure 6**). The distinct differences between the three samples, e.g., enrichment in CD3+ cells in post-COVID-19 BALF, were consistent with the results obtained from cytospins (**Supplementary Method 1**) which showed higher levels of lymphocytes in BALF 2-post-COVID-19 (11% of total cell count) as compared to the other two samples (BALF 1-non-small cell lung carcinoma: 2%, BALF 3-asthma: 1%) (**Supplementary Table 3** and **Supplementary Figure 5**; percentage of major immune cell subpopulations: **Supplementary Table 4**).

## 4 Discussion

Here we developed and tested a protocol for preparation and labeling of NP and BALF cells for analysis by MC. We found that the use of GentleMACS for tissue dissociation and of DNAse treatment in the sample preprocessing improved cell count, viability and performance of sample acquisition as compared to other methods. Using our improved protocol, we were able to detect expression of 33 major immune markers across all assessed sample types based on the commercially available MDIPA panel including additional in-house antibodies (initial gate: CD45+CD66b-; main figures: 10 commonly used immune markers; supplementary figures: additional 21 markers). Thus, we established a protocol for optimal processing of NP and BALF cells, thus setting the foundation for immune cell phenotyping by MC for future studies in airway diseases.

The immune cell composition of human airways is of great interest, as immune responses to environmental agents are playing a central role in many diseases affecting the human airways including asthma or CRS. With new techniques available, many studies currently apply single-cell RNA sequencing (19) or protein profiling (24) for the analysis of the immune response. For analysis of immune populations at a cellular level, flow cytometry is widely used both in BALF (25) and CRSwNP (26). During recent years, MC is becoming increasingly popular for deep immune profiling of PBMCs (27, 28) but its application in human non-blood derived cells especially of the human airways is still limited (12, 13). One potential reason for this is the lack of validated protocols for tissue preparation. Here we aimed to close this gap by providing a detailed, optimized, and uniform MC labeling protocol for human airway derived cells.

The first challenge in establishing the protocol was the digestion of NPs. We tested different processing protocols to identify the method for optimal preservation of cell diversity whilst thoroughly disrupting tissue structure. We found the GentleMACS™ dissociator system, which has previously been applied for the preparation of single cell suspension of NPs (29) and nasal mucosa (30) to yield optimal results in terms of cell viability and cell count. These observations are in line with other studies reporting that the automated enzymatic and mechanic GentleMACS™ method is a fast and efficient approach avoiding extensive cell disruptions, as observed with manual processing (31) whilst achieving more than 85% viability in cell suspensions (32). For some difficult to digest tissues, such as renal tumors, an additional overnight pre-digestion step with collagenase may be required to obtain single cell suspensions (33). However, as we achieved complete digestion of the polyp tissue using the commercially available kit, we did not test the effect of additional collagenase treatment in our protocol. Interestingly, protease digestion, despite yielding the lowest cell count, still preserved a higher viability of the cells than collagenase. The latter could be explained by the ability of protease to cause less cellular stress response than collagenase treatment in line with O’Flanagan et al. (34). Importantly, we observed that the GentleMACS™ based procedure was also more efficient in preserving major immune cell populations (CD3+, CD11b+ and CD45+) in comparison to collagenase and protease-based methods. Although the composition of the GentleMACS™ kit is not disclosed by the manufacturer, it can be speculated that the cocktail of digesting reagents contained in the kit has been carefully chosen and titrated, explaining the optimal performance. Furthermore, the fact that it has both mechanical and enzymatic components may also contribute to the efficiency of this protocol.

As acquisition of samples did not always run smoothly and clogging occurred frequently towards the end of the run especially when BALF was acquired, our protocol required further optimization to reduce cell debris. DNAse has been previously successfully applied for declumping of cells without affecting expression of standard markers (35). Especially for later analysis in MC, a better live cell yield has been reported by the use of collagenase in combination with DNAse as compared to collagenase alone in tissue preparations from various tissues such as melanoma or small lung cell carcinoma (36). The Basic MicroBeads have previously successfully been used for enhancing the isolation of rare cell populations by removal of dead cells (37) but also for MC protocols (38). Thus, we evaluated DNAse and Basic MicroBeads, either separately or sequentially, as methods to clean the single-cell suspensions of NP and BALF for acquisition. Whilst the three different protocols performed similar for blood and NPs, DNAse was superior in removing dead cells from BALF whilst MicroBeads seemed to damage cells as well as impeded the removal of debris in BALF leading to a lower viability. Consequently, the DNAse was incorporated as a pre-processing step before labeling for all the samples.

Once the expanded panel was ready, full labeling of blood, BALF and NP cells proceeded according to the presently established protocols. Obtained normalized data was cleaned up applying the strategy described by Bagwell et al. (22) in FCS Express™ 7 (**Supplementary Figure 2**). However, MC data analysis is not a one-size-fits-all as panels with a plethora of markers cannot be analyzed using conventional approaches assessing one marker at a time (39). Here we used Cytobank, a cloud based software that allows for a fast and uncomplicated analysis without requiring experience in programming languages such as R, Python or Matlab (3). This software offers different algorithms for dimensionality reduction as well as clustering analysis, such as viSNE and SPADE and thus provides users with an exploratory approach for high-dimensional single-cell analysis. We chose to present our data using opt-SNE, for it allowed the visualization of cell subsets present in each specimen in a reproducible way, the comparison of the cell subsets among samples and the correlation to patient conditions. Our observations corroborate the work of Belkina et al. which demonstrates that for dimensionality reduction, opt-SNE can produce better results than the other t-SNE algorithms available in Cytobank, once the parameters have been adjusted adequately (40).

In this context, it was possible to observe both the consistency of the analysis for blood and NP, as well as the differences in BALF immune cell composition according to the respective disease (**Figures 5**, **6**; **Supplementary Figures 3–5**; percentage of major cell populations obtained in NP and BALF: **Supplementary Tables 3, 4**) which are in line with previously published data: NPs showed an increased percentage of CD8+ T cells and consecutively a lower percentage of CD4+ T cells amongst CD3+ cells, as compared to PBMCs (41). The distribution of MAIT cells in PBMCs and NPs was comparable to those previously described (42). Importantly, we were also able to identify a small but distinct cell population showing characteristic features of ILC2 (Lin^-^, CD127^+^, CD294^+^, CD161^+^) at a percentage in line with work from two different groups employing a similar identification approach in MC and flow cytometry (12, 43). Furthermore, we identified a unique NK cell population expressing CD161, a marker defining most likely a pro-inflammatory subset of NK cells (44). In BALF, macrophages represent approximately 90% of the cell population (45), therefore clusters appear less separated as they present diverse activation stages and origins of the same populations (alveolar and monocyte-derived, for instance) (46). We found that our observed percentages of immune cell populations assessed with MC in BALF 1 and BALF 3 were in line first with the cell slides prepared from the identical samples (**Supplementary Table 4** and **Supplementary Figure 6**), and second with available literature reporting the preponderance of macrophages, the prevalence of CD4+ upon CD8+ T cells, and the very low and variable numbers of B cells and NK cells (13, 47–49). BALF 2 showed a strong lymphocytosis typical for COVID-19 disease (50). Furthermore, the differential expression of markers by macrophages observed *via* MC was in line with previously reported macrophage marker patterns in different airway disease entities (16, 51, 52). Thus, our data confirm that MC is well suited for identification of immune cell subsets across various tissues.

Our work has limitations, such as the relatively small number of samples for the replicates of the experiments. In this respect, it needs to be borne in mind that the goal was to optimize a protocol for preparation, acquisition, and analysis of human airway derived samples. Due to a relatively high number of cells required for MC and limited availability of samples, we could not perform flow cytometry stainings with the same antibodies in parallel, which would have been ideal for validation of our protocol. However, as reported above, the percentage of cell populations is in line with previously published observations in NP and BALF samples using flow cytometry. Furthermore, due to ethical reasons we did not include nasal mucosa biopsies or BALF from healthy volunteers, which will be of interest in future studies further characterizing the cells. This newly established protocol warrants future validation involving a representative number of patients suffering from different diseases to ensure reproducibility of the methods, ideally in a multicenter setting.

In summary, MC is an exciting method for exploration of immune subsets currently allowing for the detection of up to 60 individual markers simultaneously in a single sample (2). Our validated protocol helps to study and compare samples derived from the human airways applying MC. We lay the foundation for the comparative study of blood, airway mucosa and BALF from the patients by MC enabling in depth characterization of immune cells in diseases of upper and lower respiratory tract.

## Data availability statement

The original contributions presented in the study are included in the article/**Supplementary Material**. Further inquiries can be directed to the corresponding author.

## Ethics statement

This study was reviewed and approved by the local ethics committee (EK No. 2112/2021). Human nasal polyp (NP), bronchoalveolar lavage fluid (BALF) and blood samples were obtained from biobanks of the Department of Otorhinolaryngology and the Department of Pulmonology of the Medical University of Vienna. Written informed consent was obtained from each patient before sample submission to the biobank according to the Declaration of Helsinki and ICH GCP.

## Author contributions

JE-D, SVS, MI and MR-H designed and coordinated the study and wrote the manuscript. DG, SLS, SVS, NJC and TB obtained samples for the biobank. JE-D and MR-H performed the experiments and analysed the data. LM, AS, KK, and AW performed sample acquisition and provided input for data analysis. AZ processed the BALF and contributed to data analysis. VS, AT, and MZ provided technical support during performance of the experiments. HM provided expertise in data acquisition and analysis and critically read the manuscript. All authors contributed to the article and approved the submitted version.

## References

[1] Spitzer MH Nolan GP . Mass cytometry: Single cells, many features. Cell (2016) 165(4):780–91. doi: 10.1016/j.cell.2016.04.019 PMC4860251 27153492

[2] Akshay I Hamers AAJ Pillai AB . CyTOF® for the masses. Front Immunol (2022) 13. doi: 10.3389/fimmu.2022.815828 PMC9047695 35493491

[3] Kimball AK Oko LM Bullock BL Nemenoff RA van Dyk LF Clambey ET . A beginner’s guide to analyzing and visualizing mass cytometry data. J Immunol (2018) 200(1):3–22. doi: 10.4049/jimmunol.1701494 29255085 PMC5765874

[4] Levine JH Simonds EF Bendall SC Davis KL Amir E ad D Tadmor MD . Data-driven phenotypic dissection of AML reveals progenitor-like cells that correlate with prognosis. Cell (2015) 162(1):184–97. doi: 10.1016/j.cell.2015.05.047 PMC4508757 26095251

[5] Nair N Mei HE Chen SY Hale M Nolan GP Maecker HT . Mass cytometry as a platform for the discovery of cellular biomarkers to guide effective rheumatic disease therapy. Arthritis Res Ther (2015) 17(1):127–7. doi: 10.1186/s13075-015-0644-z PMC4436107 25981462

[6] Takahashi C Au-Yeung A Fuh F Ramirez-Montagut T Bolen CR Mathews WC . Mass cytometry panel optimization through the designed distribution of signal interference. Cytom A (2017) 91(1):39–47. doi: 10.1002/cyto.a.22977 27632576

[7] Simoni Y Chng MHY Li S Fehlings M Newell EW . Mass cytometry: a powerful tool for dissecting the immune landscape. Curr Opin Immunol (2018) 51:187–96. doi: 10.1016/j.coi.2018.03.023 29655022

[8] Zhang T Warden AR Li Y Ding X . Progress and applications of mass cytometry in sketching immune landscapes. Clin Transl Med (2020) 10(6):e206. doi: 10.1002/ctm2.206 PMC7556381 33135337

[9] Behbehani GK . Applications of mass cytometry in clinical medicine: The promise and perils of clinical CyTOF. Clin Lab Med (2017) 37(4):945–64. doi: 10.1016/j.cll.2017.07.010 29128078

[10] Tinnevelt GH Wouters K Postma G Folcarelli R Jansen JJ . High-throughput single cell data analysis – a tutorial. Anal Chim Acta (2021) 1185:338872. doi: 10.1016/j.aca.2021.338872 34711307

[11] Girodet PO Nguyen D Mancini JD Hundal M Zhou X . Alternative macrophage activation is increased in asthma. Am J Respir Cell Mol Biol (2016) 55(4):467–75. doi: 10.1165/rcmb.2015-0295OC PMC5070104 27248771

[12] Bartemes KR Choby G O’Brien EK Stokken JK Pavelko KD Kita H . Mass cytometry reveals unique subsets of T cells and lymphoid cells in nasal polyps from patients with chronic rhinosinusitis (CRS). Allergy (2020) 76(7):2222–6. doi: 10.22541/au.160081254.40990165 PMC8555365 33370459

[13] Stewart E Wang X Chupp G Montgomery RR . Profiling cellular heterogeneity in asthma with single cell multiparameter CyTOF. J Leukoc Biol (2020) 108(5):1555–64. doi: 10.1002/JLB.5MA0720-770RR PMC8087109 32911570

[14] Xu X Reitsma S Wang DY Fokkens WJ . Highlights in the advances of chronic rhinosinusitis. Allergy (2021) 76 (11):3349–58. doi: 10.1111/all.14892 33948955

[15] Yao Y Welp T Liu Q Niu N Wang X Britto CJ . Multiparameter single cell profiling of airway inflammatory cells. Cytom Part B-Clin Cytom (2017) 92(1):12–20. doi: 10.1002/cyto.b.21491 PMC5250532 27807928

[16] Dewhurst JA Lea S Hardaker E Dungwa J Ravi A Singh D . Characterisation of lung macrophage subpopulations in COPD patients and controls. Sci Rep (2017) 7(1):7143–3. doi: 10.1038/s41598-017-07101-2 PMC5540919 28769058

[17] Wahlund CJE Akpinar GG Steiner L Ibrahim A Bandeira E Lepzien R . Sarcoidosis exosomes stimulate monocytes to produce pro-inflammatory cytokines and CCL2. Sci Rep (2020) 10(1):15328–8. doi: 10.1038/s41598-020-72067-7 PMC7501276 32948789

[18] Buchheit KM Dwyer DF Ordovas-Montanes J Katz HR Lewis EI Vukovic M . IL-5Rα marks nasal polyp IgG4- and IgE-expressing cells in aspirin-exacerbated respiratory disease. J Allergy Clin Immunol (2020) 145(6):1574–84. doi: 10.1016/j.jaci.2020.02.035 PMC7282948 32199912

[19] Deprez M Zaragosi LE Truchi M Becavin C Ruiz García S Arguel MJ . A single-cell atlas of the human healthy airways. Am J Respir Crit Care Med (2020) 202(12):1636–45. doi: 10.1164/rccm.201911-2199OC 32726565

[20] Bagwell CB Hunsberger BC Hill BL Herbert DJ Bray CM Selvanantham T . Multi-site reproducibility of a human immunophenotyping assay in whole blood and peripheral blood mononuclear cells preparations using CyTOF technology coupled with maxpar pathsetter, an automated data analysis system. Cytom Part B-Clin Cytom (2020) 98(2):146–60. doi: 10.1002/cyto.b.21858 PMC7543682 31758746

[21] Maecker HT Frey T Nomura LE Trotter J . Selecting fluorochrome conjugates for maximum sensitivity. Cytom A (2004) 62(2):169–73. doi: 10.1002/cyto.a.20092 15536642

[22] Bagwell CB Inokuma M Hunsberger BC Herbert DJ Bray CM Hill BL . Automated data cleanup for mass cytometry. Cytom A (2020) 97(2):184–98. doi: 10.1002/cyto.a.23926 31737997

[23] Ioannidis LJ Mitchell AJ Zheng T Hansen DS . CyTOF mass cytometry analysis of human memory CD4+ T cells and memory b cells. STAR Protoc (2022) 3(2):101269–9. doi: 10.1016/j.xpro.2022.101269 PMC8976126 35378884

[24] Bhargava M Viken KJ Wang Q Jagtap PD Bitterman P Ingbar D . Bronchoalveolar lavage fluid protein expression in acute respiratory distress syndrome provides insights into pathways activated in subjects with different outcomes. Sci Rep (2017) 7(1):7464–4. doi: 10.1038/s41598-017-07791-8 PMC5547130 28785034

[25] Vijayakumar B Boustani K Ogger PP Papadaki A Tonkin J Christopher M . Immuno-proteomic profiling reveals aberrant immune cell regulation in the airways of individuals with ongoing post-COVD-19 respiratory disease. Immunity (2022) 55 (3):542–56.e5. doi: 10.1016/j.immuni.2022.01.017 PMC8789571 35151371

[26] Ho J Bailey M Zaunders J Mrad N Sacks R Sewell WA . Cellular comparison of sinus mucosa vs polyp tissue from a single sinus cavity in chronic rhinosinusitis. Int Forum Allergy Rhinol (2015) 5(1):14–27. doi: 10.1002/alr.21417 25332132

[27] Bengsch B Ohtani T Herati RS Bovenschen N Chang KM Wherry EJ . Deep immune profiling by mass cytometry links human T and NK cell differentiation and cytotoxic molecule expression patterns. J Immunol Methods (2017) 453:3–10. doi: 10.1016/j.jim.2017.03.009 28322863 PMC5605401

[28] Böttcher C Fernández-Zapata C Schlickeiser S Kunkel D Schulz AR Mei HE . Multi-parameter immune profiling of peripheral blood mononuclear cells by multiplexed single-cell mass cytometry in patients with early multiple sclerosis. Sci Rep (2019) 9(1):19471. doi: 10.1038/s41598-019-55852-x 31857644 PMC6923404

[29] Xu J Han R Kim DW Mo JH Jin YD Rha KS . Role of interleukin-10 on nasal polypogenesis in patients with chronic rhinosinusitis with nasal polyps. PloS One (2016) 11(9):e0161013. doi: 10.1371/journal.pone.0161013 PMC5008817 27584662

[30] Derycke L Zhang N Holtappels G Dutre T Bachert C . IL-17A as a regulator of neutrophil survival in nasal polyp disease of patients with and without cystic fibrosis. J Cyst Fibros (2012) 11(3):193–200. doi: 10.1016/j.jcf.2011.11.007 22178076

[31] Zhai X Wang W Dou D Ma Y Gang D Jiang Z . A novel technique to prepare a single cell suspension of isolated quiescent human hepatic stellate cells. Sci Rep (2019) 9(1):12757–7. doi: 10.1038/s41598-019-49287-7 PMC6726602 31485000

[32] Karmakar T Banerjee S Brahma S Dey D Radhakrishnan VS Chandy M . A pilot study to determine the utility of automated tissue dissociator for flowcytometry based evaluation of hematolymphoid tumor tissue biopsies. Indian J Hematol Blood Transfus (2021) 38 (2):1–8. doi: 10.1007/s12288-021-01481-2 35496962 PMC9001761

[33] Baldan V Griffiths R Hawkins RE Gilham DE . Efficient and reproducible generation of tumour-infiltrating lymphocytes for renal cell carcinoma. Br J Cancer (2015) 112(9):1510–8. doi: 10.1038/bjc.2015.96 PMC4453687 25867267

[34] O’Flanagan CH Campbell KR Zhang AW Kabeer F Lim JLP Biele J . Dissociation of solid tumor tissues with cold active protease for single-cell RNA-seq minimizes conserved collagenase-associated stress responses. Genome Biol (2019) 20(1):1–13. doi: 10.1186/s13059-019-1830-0 31623682 PMC6796327

[35] García-Piñeres AJ Hildesheim A Williams M Trivett MT Strobl S Pinto LA . DNAse treatment following thawing of cryopreserved PBMC is a procedure suitable for lymphocyte functional studies. J Immunol Methods (2006) 313(1):209–13. doi: 10.1016/j.jim.2006.04.004 16737707

[36] Leelatian N Doxie DB Greenplate AR Mobley BC Lehman JM Sinnaeve J . Single cell analysis of human tissues and solid tumors with mass cytometry. Cytom Part B - Clin Cytom (2017) 92(1):68–78. doi: 10.1002/cyto.b.21481 PMC5459378 27598832

[37] Lin D Maecker HT . Mass cytometry assays for antigen-specific T cells using CyTOF. Methods Mol Biol (2018) 1678:37–47. doi: 10.1007/978-1-4939-7346-0_3 29071674 PMC5798871

[38] Lee MYC Lufkin T . Development of the "Three-step MACS": a novel strategy for isolating rare cell populations in the absence of known cell surface markers from complex animal tissue. J Biomol Tech JBT (2012) 23 (2):69–77. doi: 10.7171/jbt.12-2302-003 PMC3336840 22951961

[39] Marsh-Wakefield F Mitchell AJ Norton SE Ashhurst TM Leman JK Roberts JM . Making the most of high-dimensional cytometry data. Immunol Cell Biol (2021) 99(7):680–96. doi: 10.1111/imcb.12456 PMC8453896 33797774

[40] Belkina AC Ciccolella CO Anno R Halpert R Spidlen J Snyder-Cappione JE . Automated optimized parameters for T-distributed stochastic neighbor embedding improve visualization and analysis of large datasets. Nat Commun (2019) 10(1):5415–5. doi: 10.1038/s41467-019-13055-y PMC6882880 31780669

[41] Ickrath P Kleinsasser N Ding X Ginzkey C Beyersdorf N Hagen R . Characterization of T-cell subpopulations in patients with chronic rhinosinusitis with nasal polyposis. Allergy Rhinol Provid RI (2017) 8(3):139–47. doi: 10.2500/ar.2017.8.0214 PMC5662539 29070271

[42] Rha MS Yoon YH Koh JY Jung JH Lee HS Park SK . IL-17A-producing sinonasal MAIT cells in patients with chronic rhinosinusitis with nasal polyps. J Allergy Clin Immunol (2022) 149 (2):599–609. doi: 10.1016/j.jaci.2021.07.037 34403659

[43] Poposki JA Klingler AI Tan BK Soroosh P Banie H Lewis G . Group 2 innate lymphoid cells are elevated and activated in chronic rhinosinusitis with nasal polyps. Immun Inflammation Dis (2017) 5(3):233–43. doi: 10.1002/iid3.161 PMC5569375 28474861

[44] Kurioka A Cosgrove C Simoni Y Wilgenburg B Geremia A Björkander S . CD161 defines a functionally distinct subset of pro-inflammatory natural killer cells. Front Immunol (2018) 9:486–6. doi: 10.3389/fimmu.2018.00486 PMC5900032 29686665

[45] Joshi N Walter JM Misharin AV . Alveolar macrophages. Cell Immunol (20180 330:86–90. doi: 10.1016/j.cellimm.2018.01.005 29370889

[46] Yu YRA Hotten DF Malakhau Y Volker E Ghio AJ Noble PW . Flow cytometric analysis of myeloid cells in human blood, bronchoalveolar lavage, and lung tissues. Am J Respir Cell Mol Biol (2016) 54(1):13–24. doi: 10.1165/rcmb.2015-0146OC 26267148 PMC4742930

[47] Harbeck RJ . Immunophenotyping of bronchoalveolar lavage lymphocytes. Clin Vaccine Immunol (1998) 5(3):271–7. doi: 10.1128/CDLI.5.3.271-277.1998 PMC104508 9605975

[48] Heron M Grutters JC Dam-Molenkamp KMt Hijdra D van Heugten-Roeling A Claessen AM . Bronchoalveolar lavage cell pattern from healthy human lung. Clin Exp Immunol (2012) 167(3):523–31. doi: 10.1111/j.1365-2249.2011.04529.x PMC3374285 22288596

[49] d’Alessandro M Bergantini L Mezzasalma F Cavallaro D Gangi S Baglioni S . Immune-checkpoint expression on CD4, CD8 and NK cells in blood, bronchoalveolar lavage and lymph nodes of sarcoidosis. Mol Diagn Ther (2022) 26 (4):437–49. doi: 10.1183/23120541.LSC-2022.155 PMC9276617 35761164

[50] Gelarden I Nguyen J Gao J Chen Q Morales-Nebreda L Wunderink RG . Comprehensive evaluation of bronchoalveolar lavage from patients with severe COVID-19 and correlation with clinical outcomes. Hum Pathol (2021) 113:92–103. doi: 10.1016/j.humpath.2021.04.010 33905777 PMC8159767

[51] Allard B Panariti A Martin JG . Alveolar macrophages in the resolution of inflammation, tissue repair, and tolerance to infection. Front Immunol (2018) 9. doi: 10.3389/fimmu.2018.01777 PMC6079255 30108592

[52] Rendeiro AF Ravichandran H Bram Y Chandar V Kim J Meydan C . The spatial landscape of lung pathology during COVID-19 progression. Nature (2021) 593(7860):564–9. doi: 10.1038/s41586-021-03475-6 PMC8204801 33780969

