# Supplementary material for: Using mass cytometry for the analysis of samples of the human airways
Source: Front Immunol. 2022 Dec 12;13:1004583. doi: 10.3389/fimmu.2022.1004583 (PMC9791368; doi:10.3389/fimmu.2022.1004583)
Supplement: Supplementary file 1 [file DataSheet_1.pdf]

Supplementary Material

Methods

1. Cytospins

Two cytospins (Shandon Cytospin 3 (ThermoFisher Scientific)) were prepared for each BALF sample. Briefly, 2.5 x 10^5 BALF cells were washed with cold PBS (Gibco, Thermo Fisher Scientific) twice before resuspending the cells in 400 µL PBS supplemented with 1% BSA (Sigma Aldrich). The slides, filters, and single cytology funnels (Biomedical Polymers, Jacksonville, Florida, USA) were placed into the slots of the cytospin (Rotofix 32 A, Hettich, Tuttlingen, Germany), and 200 µL cell suspension (1.25 x 10^5 cells) were loaded into a funnel. Cytospin was set on 900 rpm for 8 min at room temperature (RT). Subsequently, slides were stained by incubating them in May-Gruenwald solution (Sigma Aldrich) for 9 min. Afterwards slides were washed with water and incubated in Giemsa solution (Sigma Aldrich) for another 9 min. Slides were washed with water again and mounted with Eukitt mounting medium (Sigma Aldrich).

2. Mass Cytometry Staining Protocols

*Note: do not use latex gloves or glass wear. Use only propylene tubes and filtered tips. For information on manufacturer of the reagents please refer to the main methods part of this paper.

Whole Blood

⏱ **Timing: about 4 h**

1.) Work with fresh blood collected in a heparin tube.
2.) Prepare fresh 0.01 % DNAse solution in Maxpar Cell Staining Buffer (CSB).
3.) Resuspend 400 µL of heparinised blood with 1 mL DNAse solution in a 1.5 mL Eppendorf tube.
4.) Seal and incubate at 37 °C x 30 min x 300 rpm.
5.) Transfer to a sterile polypropylene 5 mL round-bottom polypropylene test tube, add 1 mL of CSB, mix well, and centrifuge at 300 g x 7 min x RT.
6.) Remove the supernatant, resuspend the pellet in 1 mL of CSB supplemented with 10 µL of 10 KU/mL heparin solution.
7.) Incubate for 20 min x RT.
8.) In the meantime, prepare the mix of additional antibodies for a final volume of 30 µL according to the table below, to be used in step 13.

Antibody	Metal tag	Dilution Antibody
CD11b/Mac-1	^{209}Bi	1/200
CD23	^{159}Tb	1/400
FcεRI	^{116}Cd	1/200
**Complete with CSB up to the final volume of 30 µL**		

9.) After step 7, add 2 mL of CSB to the sample tube. Mix well by pipetting.
10.) Centrifuge at 300 g x 7 min x RT.
11.) Remove the supernatant and resuspend in 270 µL of CSB.
12.) Open the Maxpar antibody pellet (provided in the kit), add the full volume of resuspended cells to it and mix well by pipetting.
13.) Add the antibody mix prepared in step 8 and mix well by pipetting.
14.) Incubate for 30 min x RT.
15.) Add 250 µL of Cal-Lyse lysing solution and mix well by pipetting.
16.) Incubate in the dark for 10 min x RT.
17.) Add 3 mL of de-ionized water and mix well by pipetting.
18.) Incubate in the dark for 10 min x RT.
   *Note: if the solution is not translucent, incubate in the dark for another 5 min x RT.
19.) Centrifuge at 300 g x 7 min x RT.
20.) Remove the supernatant and resuspend the cells in 3 mL of CSB. Mix well by pipetting.
21.) Centrifuge at 300 g x 7 min x RT.
22.) Repeat steps 20-21.
23.) During the last centrifugation, prepare a fresh 1.6 % formaldehyde solution in Maxpar PBS.
24.) Remove the supernatant and resuspend the cells in 1 mL of the 1.6 % formaldehyde solution. Mix well by pipetting.
25.) Incubate for 10 min x RT.
26.) Centrifuge at 800 g x 7 min x RT.
27.) In the meantime, prepare the DNA Intercalation Solution: 1 mL of FIX PERM Buffer + 1 µL of Intercalator-IR.
28.) Remove the supernatant and resuspend the cells in 1 mL of Intercalation Solution. Mix well by pipetting.
29.) Incubate for 30 min x RT.
30.) Meanwhile, count the cells.
31.) Centrifuge at 800 g x 7 min x RT.
32.) Aspirate 900 µL of the supernatant and resuspend the pellet in the residual volume (100 µL).
33.) Store at -80°C in a cell freezing container for 24h, then transfer to a sample box at -80°C.
   *Note: the samples can be stored up to 4 months!

Nasal Polyp

Timing: about 5 h

1.) Work with fresh nasal polyp tissue from surgery or biopsy.
2.) Prepare the reagents of the "Multi Tissue Dissociation Kit 1" (Miltenyi) following the manufacturer's protocol.
   *Note: prepare the reagents in advance and keep aliquots of the enzymes in a -20 ° C freezer.
3.) Mix 2.35 mL of serum-free MEM supplemented with 1 % Penicilin/Streptomycin (MEM/PS), 100 µL of Enzyme D, 50 µL of Enzyme R, and 12.5 µL of Enzyme A of the Multi Tissue Dissociation Kit 1 into a GentleMACS C tube.
4.) Rinse the nasal polyp with Maxpar PBS in a petri dish, weigh the specimen and separate 0.5 g for use in this protocol.
5.) Place it back into the petri-dish and mince it with a scalpel.

6.) Transfer the pieces to the GentleMACS C tube prepared on step 2.
7.) Make sure the tube is well closed and attach it to the GentleMACS dissociator.
8.) Run the program "37C_Multi_B" (it takes 61 min).
9.) Remove the tube from the dissociator and resuspend the cell suspension by pipetting. If it is too thick, add 1 or 2 mL of MEM/PS by rinsing the cones of the tube and mix well.
10.) Pass the cell suspension through a MACS SmartStrainer of 30 µm mesh with the help of a 20 mL syringe stopper and collect it in a 50 mL conical tube.
11.) Wash the Smart Strainer with 15 mL of MEM/PS and add fetal bovine serum to a final concentration of 10%.
*Note: discount 1 or 2 mL of MEM/PS in case it was added on step 8!
12.) Mix well and centrifuge at 300 g x 7 min x RT.
13.) Meanwhile, prepare fresh 0.01 % DNAse solution in Maxpar CSB.
14.) Remove the supernatant of the nasal polyp cell suspension and resuspend the pellet in 1 mL of DNase solution in a 1.5 mL Eppendorf tube.
15.) Seal and incubate at 37 °C x 30 min x 300 rpm.
16.) Transfer to a sterile polypropylene 5 mL round-bottom polypropylene test tube, add 1 mL of CSB, mix well, and centrifuge at 300 g x 7 min x RT.
17.) Remove the supernatant, resuspend the cells in 1 mL of CSB supplemented with 10 µL of 10 KU/mL heparin solution.
*Note: count cells! (The suspension should contain around 1 – 3 x 10^6 cells)
18.) Incubate for 20 min x RT.
19.) Centrifuge at 300 g x 7 min x RT.
20.) Remove the supernatant and resuspend the cells in 50 µL of CSB and 5 µL of Human TruStain FcX. Mix well by pipetting.
21.) Incubate for 10 min x RT.
22.) In the meantime, prepare the mix of additional antibodies for a final volume of 30 µL according to the table below, to be used in step 25.

Antibody	Metal tag	Dilution Antibody
CD11b/Mac-1	^{209}Bi	1/200
CD23	^{159}Tb	1/200
FcεRI	^{116}Cd	1/400
**Complete with CSB up to the final volume of 30 µL**		

23.) After step 21, add 215 µL of CSB to the sample tube. Mix well by pipetting.
24.) Open the Maxpar antibody pellet (provided in the kit), add the full volume of resuspended cells to it and mix well by pipetting.
25.) Add the antibody mix prepared in step 22 and mix well by pipetting.
26.) Incubate for 30 min x RT.
27.) Add 3 mL of CSB, mix well by pipetting and centrifuge at 300 g x 7 min x RT.
28.) Remove the supernatant and repeat step 27.
29.) During the last centrifugation, prepare a fresh 1.6 % formaldehyde solution in Maxpar PBS.
30.) Remove the supernatant and resuspend the cells in 1 mL of the 1.6 % formaldehyde solution. Mix well by pipetting.
31.) Incubate for 10 min x RT.
32.) Centrifuge at 800 g x 7 min x RT.

33.) In the meantime, prepare the DNA Intercalation Solution: 1 mL of FIX PERM Buffer + 1 µL of Intercalator-IR.
34.) Remove the supernatant.
35.) Resuspend the cells in 1 mL of Intercalation Solution and mix well by pipetting.
36.) Incubate for 30 min x RT.
37.) Meanwhile, count the cells.
38.) Centrifuge at 800 g x 7 min x RT.
39.) Aspirate 900 µL of the supernatant and resuspend the pellet in the residual volume (100 µL).
40.) Store at -80°C in a cell freezing container for 24h, then transfer to a sample box at -80°C.
*Note: the samples can be stored up to 4 months!

Bronchoalveolar Lavage Fluid (BALF)

⏱ **Timing: about 4 h**

1.) Work with fresh BALF. Count the cells. Perform the protocol with 1 – 3 x 10^6 cells/mL.
2.) Centrifuge at 300 g x 7 min x RT.
3.) Meanwhile, prepare fresh 0.01 % DNAse solution in Maxpar CSB.
4.) Remove the supernatant of the cell suspension and resuspend the pellet in 1 mL of DNase solution in a 1.5 mL Eppendorf tube.
5.) Seal and incubate at 37 °C x 30 min x 300 rpm.
6.) Transfer to a sterile polypropylene 5 mL round-bottom polypropylene test tube, add 1 mL of CSB, mix well, and centrifuge at 300 g x 7 min x RT.
7.) Remove the supernatant, resuspend the cells in 1 mL of CSB supplemented with 10 µL of 10 KU/mL heparin solution.
8.) Incubate for 20 min x RT.
9.) Centrifuge at 300 g x 7 min x RT.
10.) Remove the supernatant and resuspend the cells in 50 µL of CSB and 5 µL of Human TruStain FcX. Mix well by pipetting.
11.) Incubate for 10 min x RT.
12.) In the meantime, prepare the mix of additional antibodies for a final volume of 30 µL according to the table below, to be used in step 15.

**Antibody**	**Metal tag**	**Dilution Antibody**
CD11b/Mac-1	^{209}Bi	1/200
CD23	^{159}Tb	1/200
FcεRI	^{116}Cd	1/400
**Complete with CSB up to the final volume of 30 µL**		

13.) After step 11, add 215 µL of CSB to the sample tube. Mix well by pipetting.
14.) Open the Maxpar antibody pellet (provided in the kit), add the full volume of resuspended cells to it and mix well by pipetting.
15.) Add the antibody mix prepared in step 12 and mix well by pipetting.
16.) Incubate for 30 min x RT.
17.) Add 3 mL of CSB, mix well by pipetting and centrifuge at 300 g x 7 min x RT.
18.) Remove the supernatant and repeat step 17.

19.) During the last centrifugation, prepare a fresh 1.6 % formaldehyde solution in Maxpar PBS.
20.) Remove the supernatant and resuspend the cells in 1 mL of the 1.6 % formaldehyde solution. Mix well by pipetting.
21.) Incubate for 10 min x RT.
22.) Centrifuge at 800 g x 7 min x RT.
23.) In the meantime, prepare the DNA Intercalation Solution: 1 mL of FIX PERM Buffer + 1 µL of Intercalator-IR.
24.) Remove the supernatant.
25.) Resuspend the cells in 1 mL of Intercalation Solution and mix well by pipetting.
26.) Incubate for 30 min x RT.
27.) Meanwhile, count the cells.
28.) Centrifuge at 800 g x 7 min x RT.
29.) Aspirate 900 µL of the supernatant and resuspend the pellet in the residual volume (100 µL).
30.) Store at -80°C in a cell freezing container for 24h, then transfer to a sample box at -80°C.
*Note: the samples can be stored up to 4 months!

Supplementary Table 1: Mass cytometry panel of antibodies and parameters for standardization experiments.

Metal	Antibody/Parameter	Clone	Manufacturer
**^{112}Cd**	CD45	HI30	Fluidigm
**^{154}Sm**	CD3	UCHT1	Fluidigm
**^{209}Bi**	CD11b/Mac-1	ICRF44	Fluidigm
**^{103}Rh**	DNA intercalator (for dead cell detection)	-	Fluidigm
**^{191}Ir** **^{193}Ir**	DNA intercalator (for nucleated cell detection)	-	Fluidigm

Supplementary Table 2: Modified Maxpar Direct Immune Profiling Assay (MMDIPA) antibody panel for mass cytometry

Metal	Antibody	Clone	Manufacturer
^{89}**Y**	CD45	HI30	Fluidigm
^{116}**Cd**	FcεRIα	AER-37 (CRA-1)	Biolegend (in-house)
^{141}**Pr**	CD196 (CCR6)	G034E3	Fluidigm
^{143}**Nd**	CD123 (IL-3R)	6H6	Fluidigm
^{144}**Nd**	CD19	HIB19	Fluidigm
^{145}**Nd**	CD4	RPA-T4	Fluidigm
^{146}**Nd**	CD8a	RPA-T8	Fluidigm
^{147}**Sm**	CD11c	Bu15	Fluidigm
^{148}**Nd**	CD16	3G8	Fluidigm
^{149}**Sm**	CD45RO	UCHL1	Fluidigm
^{150}**Nd**	CD45RA	HI100	Fluidigm
^{151}**Eu**	CD161	HP-3G10	Fluidigm
^{152}**Sm**	CD194/CCR4	L291H4	Fluidigm
^{153}**Eu**	CD25	BC96	Fluidigm
^{154}**Sm**	CD27	O323	Fluidigm
^{155}**Gd**	CD57	HCD57	Fluidigm
^{156}**Gd**	CD183 (CXCR3)	G025H7	Fluidigm

**^{158}Gd**	CD185 (CXCR5)	J252D4	Fluidigm
**^{159}Tb**	CD23	EBVCS-5	Biolegend (in-house)
**^{160}Gd**	CD28	CD28.2	Fluidigm
**^{161}Dy**	CD38	HB-7	Fluidigm
**^{163}Dy**	CD56 (NCAM)	NCAM16.2	Fluidigm
**^{164}Dy**	TCRgd	B1	Fluidigm
**^{166}Er**	CD294 (CRTH2)	BM16	Fluidigm
**^{167}Er**	CD197 (CCR7)	G043H7	Fluidigm
**^{168}Er**	CD14	63D3	Fluidigm
**^{170}Er**	CD3	UCHT1	Fluidigm
**^{171}Yb**	CD20	2H7	Fluidigm
**^{172}Yb**	CD66b	G10F5	Fluidigm
**^{173}Yb**	HLA-DR	LN3	Fluidigm
**^{174}Yb**	IgD	IA6-2	Fluidigm
**^{176}Yb**	CD127 (IL-7Ra)	A019D5	Fluidigm
**^{209}Bi**	CD11b	ICRF44	Fluidigm

Supplementary Table 3: Mean percentages of major cell populations gated in whole blood and nasal polyp.

	Mean percentage of gated cells + Standard deviation (%)			
**Samples**	**B cells**	**CD4+ T cells**	**CD8+ T cells**	**Monocytes (Blood) / Macrophages (Nasal Polyp)**
**Blood**	5.5 ± 2.4	40.3 ± 7.9	16.1 ± 8.7	18.1 ± 2.7
**Nasal Polyp**	8.9 ± 7.2	20.5 ± 9.6	24.8 ± 10.3	13.7 ± 7.3

Supplementary Table 4: Percentages of major cell populations gated in three bronchoalveolar lavage fluid samples (BALFs).

	Percentage of gated cells (%)			
**BALF samples**	**B cells**	**CD4+ T cells**	**CD8+ T cells**	**Macrophages**
**BALF 1**	0	0.3	0.1	98.7
**BALF 2**	9.1	29.2	5.6	48.6
**BALF 3**	0.1	0.2	0.1	98.1

BALF 1: non-small cell lung carcinoma; BALF 2: post-COVID-19; BALF 3: asthma.

Supplementary Table 5: Proportion of major cell populations in three bronchoalveolar lavage fluid samples (BALFs), obtained from cytospins.

	**Cell count (%)**		
**BALF samples**	**Macrophages**	**Lymphocytes**	**Eosinophils**
**BALF 1**	98	2	0
**BALF 2**	84	11	5
**BALF 3**	98	1	1

BALF 1: non-small cell lung carcinoma; BALF 2: post-COVID-19; BALF 3: asthma.

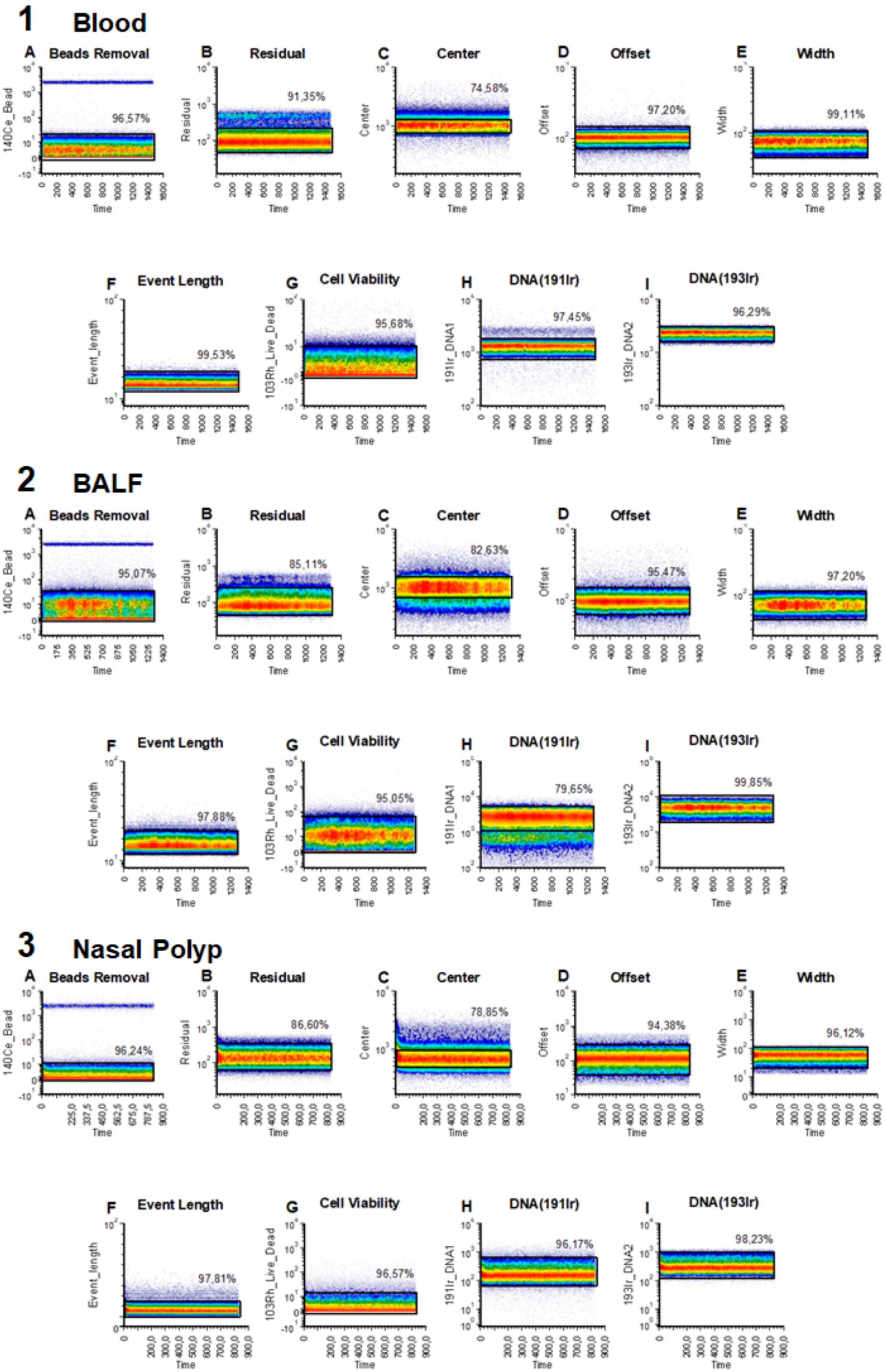
1 Blood
A Beads Removal
96,57%
B Residual
91,35%
C Center
74,58%
D Offset
97,20%
E Width
99,11%
F Event Length
99,53%
G Cell Viability
95,68%
H DNA(191Ir)
97,45%
I DNA(193Ir)
96,29%
2 BALF
A Beads Removal
95,07%
B Residual
85,11%
C Center
82,63%
D Offset
95,47%
E Width
97,20%
F Event Length
97,88%
G Cell Viability
95,05%
H DNA(191Ir)
79,65%
I DNA(193Ir)
99,85%
3 Nasal Polyp
A Beads Removal
96,24%
B Residual
86,60%
C Center
78,85%
D Offset
94,38%
E Width
96,12%
F Event Length
97,81%
G Cell Viability
96,57%
H DNA(191Ir)
96,17%
I DNA(193Ir)
98,23%
140Ce_Bead
Residual
Center
Offset
Width
Event_length
103Rh_Live_Dead
191Ir_DNA1
193Ir_DNA2
Time

Supplementary Figure 1: Mass cytometry data clean up by manual gating. The biaxial plots represent one of the three independent experiments performed with (1) blood, (2) bronchoalveolar lavage fluid - BALF and (3) nasal polyp cells. The following parameters (A) beads, (B) residual, (C) center, (D) offset, (E) width, (F) event length, and (G, H, I) DNA intercalator signals were plotted against the time of sample acquisition in minutes by gaussian discrimination as previously described (23). The plots were created with FCS Express^{TM} 7.

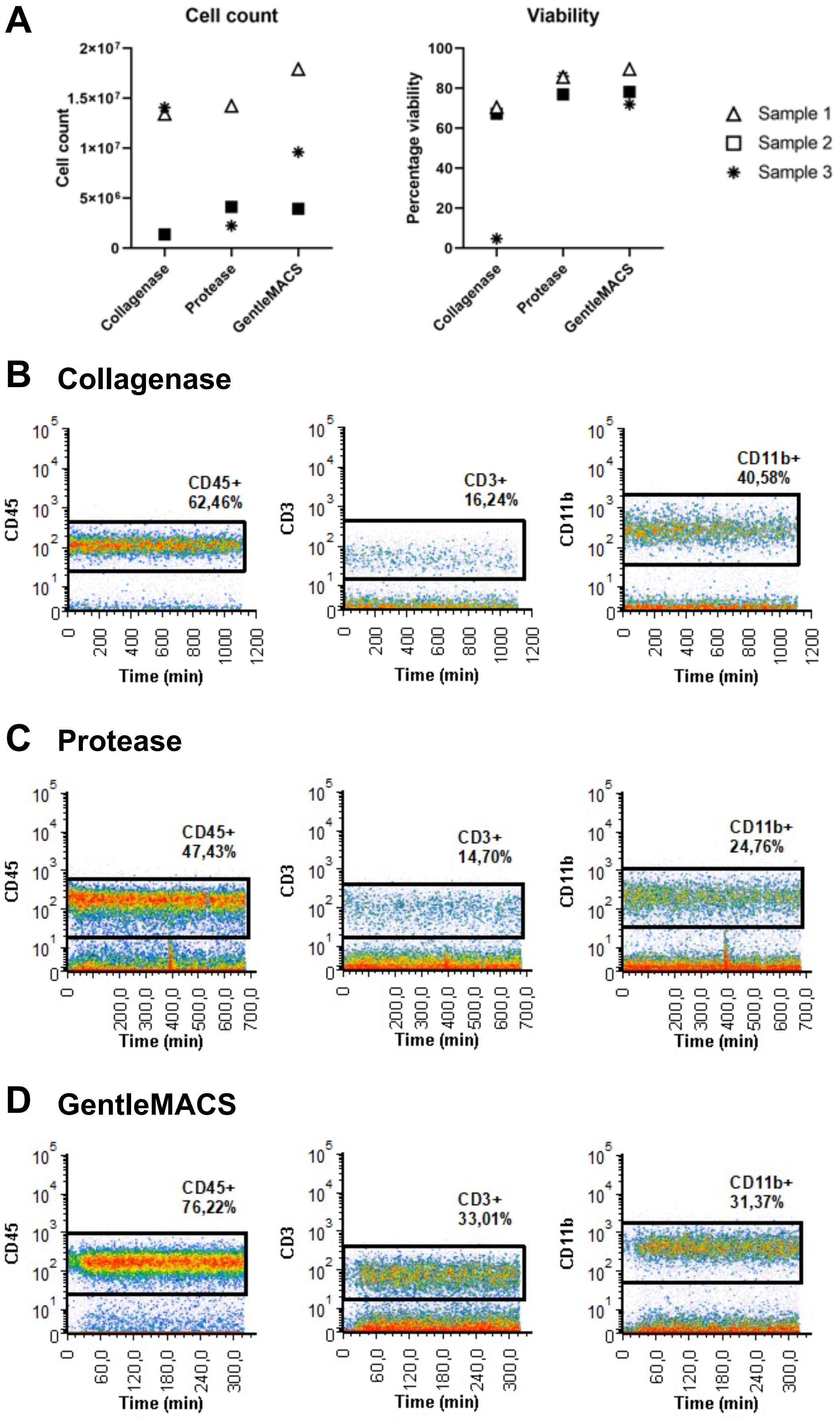
A
Cell count
Viability
Cell count
Percentage viability
Collagenase
Protease
GentleMACS
Sample 1
Sample 2
Sample 3
B Collagenase
CD45+ 62,46%
CD3+ 16,24%
CD11b+ 40,58%
C Protease
CD45+ 47,43%
CD3+ 14,70%
CD11b+ 24,76%
D GentleMACS
CD45+ 76,22%
CD3+ 33,01%
CD11b+ 31,37%
CD45
CD3
CD11b
Time (min)

Supplementary Figure 2: Nasal polyp digestion protocols for mass cytometry. (A) Mean of the cell count after digestion (total number, y-axis) and cellular viability upon acquisition by mass cytometry (given in percentage, y-axis) obtained by the three different digestion protocols (x-axis). (B, C, D) Representative dot plots of nasal polyp cells stained with major immune cell markers anti-CD45, anti-CD3 and anti-CD11b by mass cytometry. The cells were obtained from tissue processed using three different protocols: (B) collagenase, (C) protease, and (D) enzymatic/mechanical dissociation with GentleMACS. The biaxial plots represent one of the three independent experiments of each protocol performed, and the immune cell markers (Y axes) are plotted against the time of the acquisition of the sample in minutes (X axes). The plots were created with FCS Express^{TM} 7.

Blood

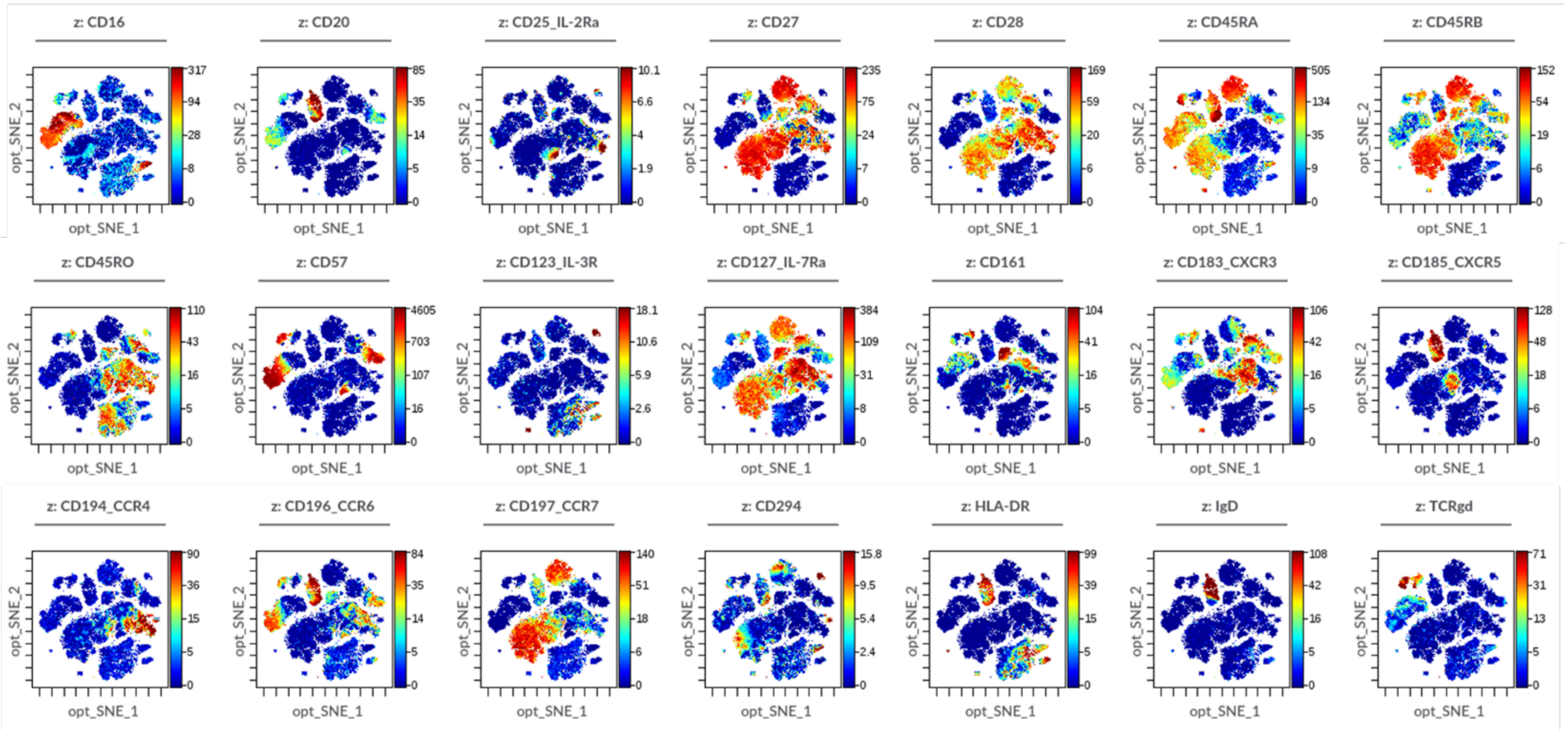
z: CD16
z: CD20
z: CD25_IL-2Ra
z: CD27
z: CD28
z: CD45RA
z: CD45RB
z: CD45RO
z: CD57
z: CD123_IL-3R
z: CD127_IL-7Ra
z: CD161
z: CD183_CXCR3
z: CD185_CXCR5
z: CD194_CCR4
z: CD196_CCR6
z: CD197_CCR7
z: CD294
z: HLA-DR
z: IgD
z: TCRgd
opt_SNE_1
opt_SNE_2

Supplementary Figure 3: Immune cell markers in human blood. Opt-SNE plots show selected markers of the modified Maxpar Direct Immune Profiling Assay (MMDIPA panel), namely CD16, CD20, CD25/IL-R2a, CD27, CD28, CD45RA, CD45RB, CD45RO, CD57, CD123/IL-3R, CD127/IL-7Ra, CD161, CD183/CXCR3, CD185/CXCR5, CD194/CCR4, CD196/CCR6, CD197/CCR7, CD294, HLA-DR, IgD, and TCRgd in human blood single cell suspension acquired in Helios® (Standard BioTools). The scales vary throughout the plots according to the intensity presented by the markers. Plots shown are representative of three independent experiments.

Nasal Polyp

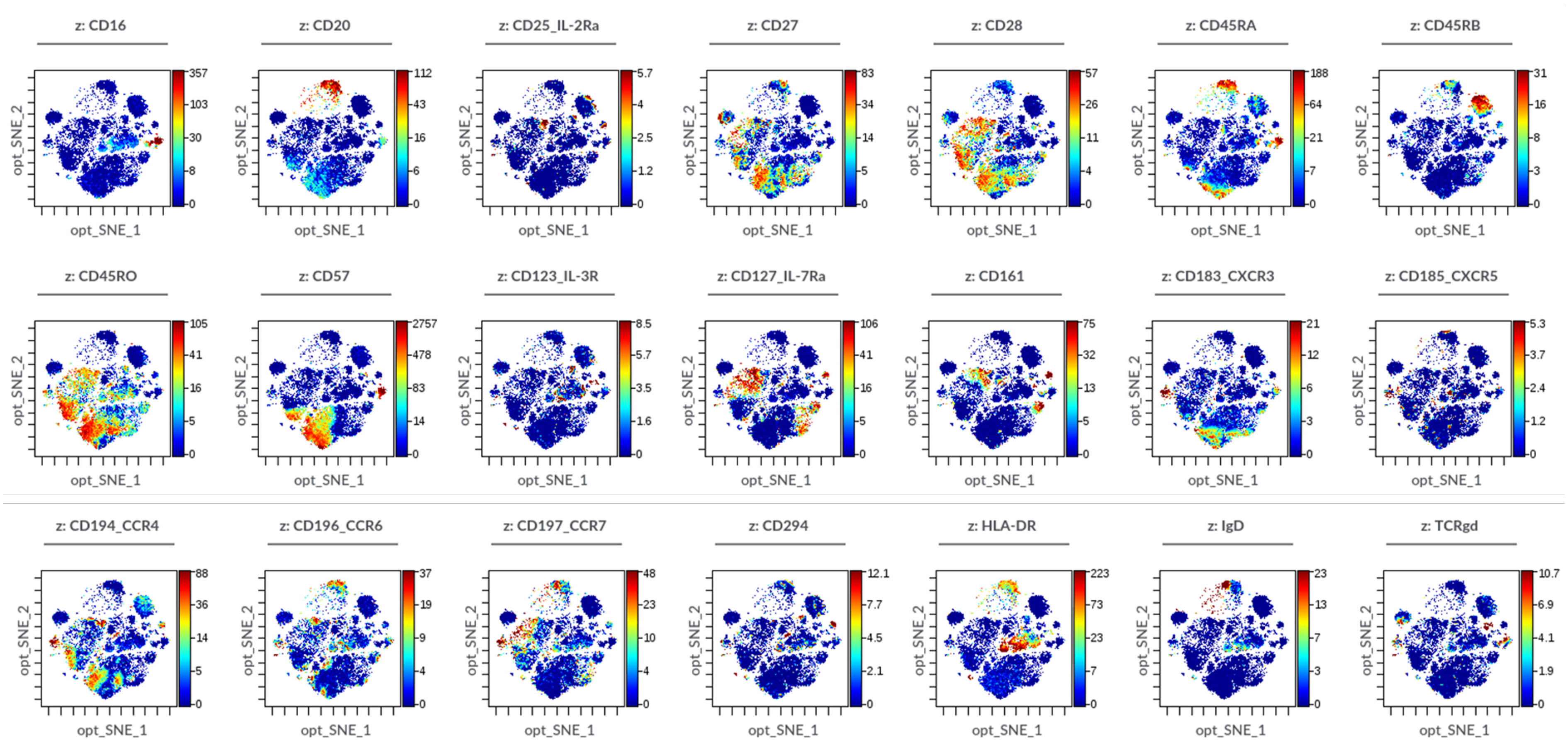
z: CD16
z: CD20
z: CD25_IL-2Ra
z: CD27
z: CD28
z: CD45RA
z: CD45RB
z: CD45RO
z: CD57
z: CD123_IL-3R
z: CD127_IL-7Ra
z: CD161
z: CD183_CXCR3
z: CD185_CXCR5
z: CD194_CCR4
z: CD196_CCR6
z: CD197_CCR7
z: CD294
z: HLA-DR
z: IgD
z: TCRgd
opt_SNE_1
opt_SNE_2

Supplementary Figure 4: Immune cell markers in human nasal polyp. Opt-SNE plots show selected markers of the modified Maxpar Direct Immune Profiling Assay (MMDIPA panel), namely CD16, CD20, CD25/IL-R2a, CD27, CD28, CD45RA, CD45RB, CD45RO, CD57, CD123/IL-3R, CD127/IL-7Ra, CD161, CD183/CXCR3, CD185/CXCR5, CD194/CCR4, CD196/CCR6, CD197/CCR7, CD294, HLA-DR, IgD, and TCRgd in human nasal polyp single cell suspension acquired in Helios® (Standard BioTools). The scales vary throughout the plots according to the intensity presented by the markers. Plots shown are representative of three independent experiments.

BALF

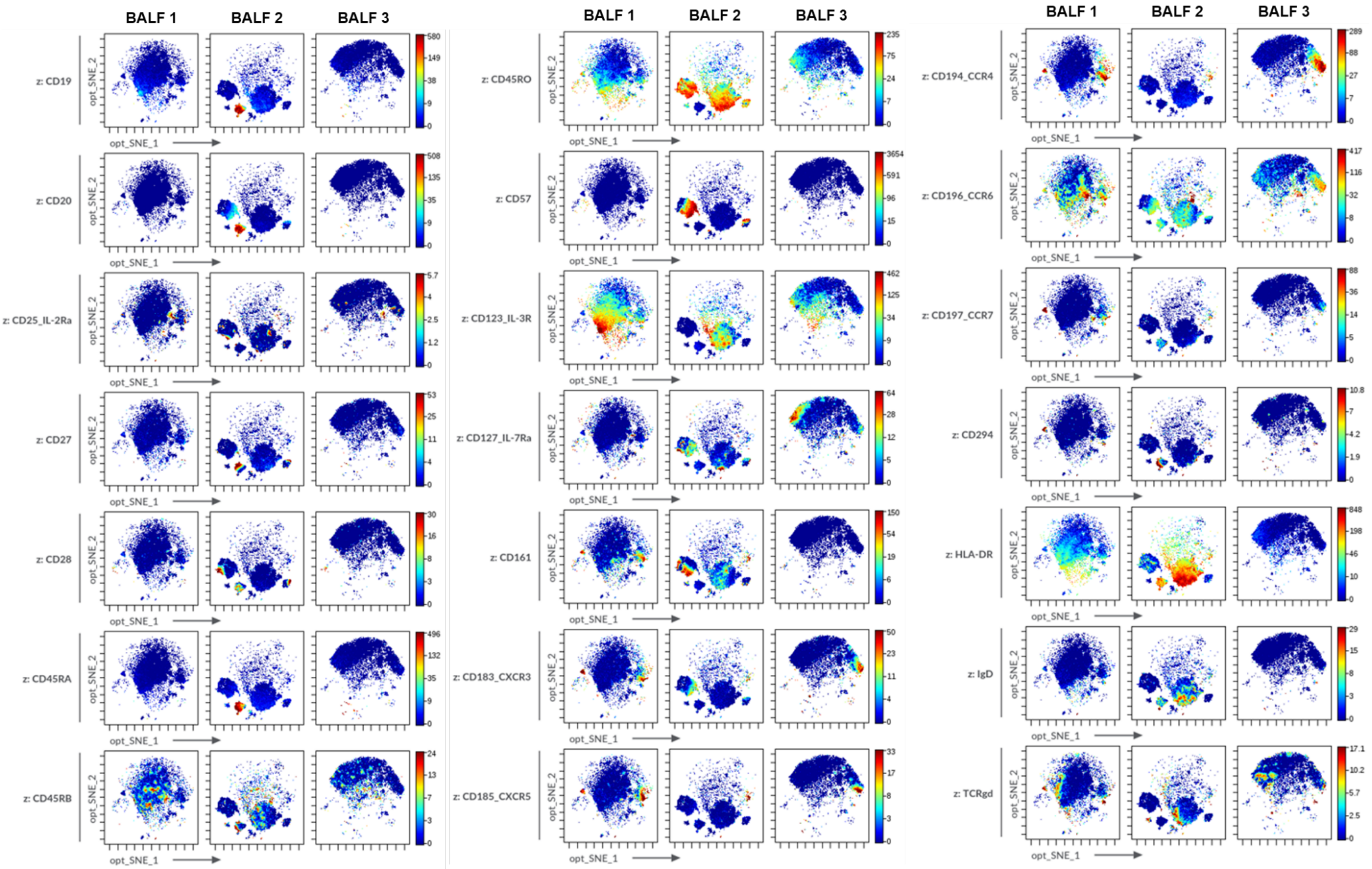

BALF 1
BALF 2
BALF 3
opt_SNE_1
opt_SNE_2
z: CD19
z: CD20
z: CD25_IL-2Ra
z: CD27
z: CD28
z: CD45RA
z: CD45RB
z: CD45RO
z: CD57
z: CD123_IL-3R
z: CD127_IL-7Ra
z: CD161
z: CD183_CXCR3
z: CD185_CXCR5
z: CD194_CCR4
z: CD196_CCR6
z: CD197_CCR7
z: CD294
z: HLA-DR
z: IgD
z: TCRgd

Supplementary Figure 5: Immune cell markers in human bronchoalveolar lavage fluid (BALF) of diverse patients acquired in Helios® (Standard BioTools). Opt-SNE plots show selected markers of the modified Maxpar Direct Immune Profiling Assay (MMDIPA panel) namely CD19, CD20, CD25/IL-R2a, CD27, CD28, CD45RA, CD45RB, CD45RO, CD57, CD123/IL-3R, CD127/IL-7Ra, CD161, CD183/CXCR3, CD185/CXCR5, CD194/CCR4, CD196/CCR6, CD197/CCR7, CD294, HLA-DR, IgD, and TCRgd. BALFs from patients with (A): non-small cell lung carcinoma (BALF 1), (B) previous COVID-19 infection (BALF 2) and (C) asthma (BALF 3) are shown. Plots shown are representative of three independent experiments.

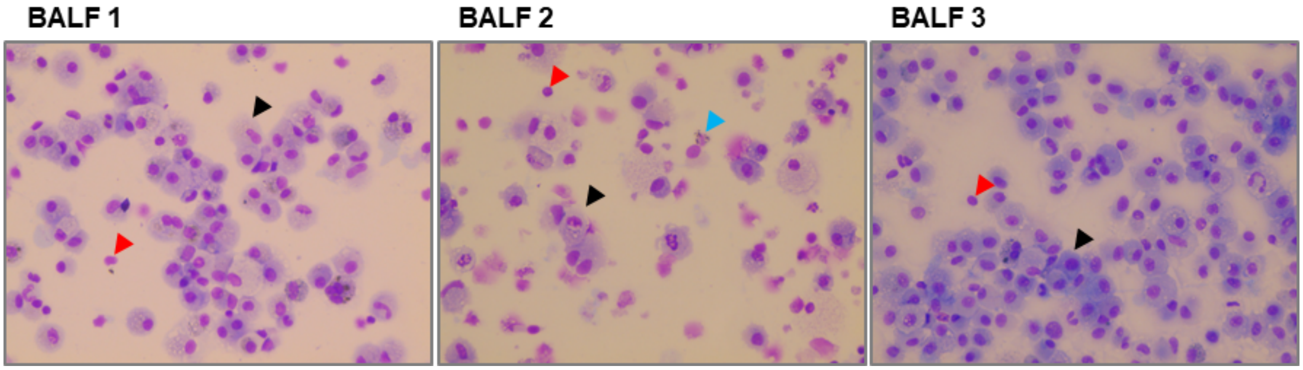

Supplementary Figure 6: H&E staining of bronchoalveolar lavage fluids (BALFs) obtained for mass cytometry analysis. Red arrow: lymphocytes. Black arrow: macrophages. Blue arrow: eosinophil. Magnification 40 X.
